# Misfolding of fukutin-related protein (FKRP) variants in congenital and limb girdle muscular dystrophies

**DOI:** 10.3389/fmolb.2023.1279700

**Published:** 2023-12-07

**Authors:** Christopher T. Esapa, R. A. Jeffrey McIlhinney, Adrian J. Waite, Matthew A. Benson, Jasmin Mirzayan, Henriett Piko, Ágnes Herczegfalvi, Rita Horvath, Veronika Karcagi, Maggie C. Walter, Hanns Lochmüller, Pierre J. Rizkallah, Qi L. Lu, Derek J. Blake

**Affiliations:** ^1^ MRC Harwell Institute, Harwell Campus, Oxfordshire, United Kingdom; ^2^ MRC Anatomical Neuropharmacology Unit, University of Oxford, Oxford, United Kingdom; ^3^ Centre for Neuropsychiatric Genetics and Genomics, Division of Psychological Medicine and Clinical Neurosciences, School of Medicine, Cardiff University, Cardiff, United Kingdom; ^4^ Charles River Laboratories, Saffron Walden, United Kingdom; ^5^ Department of Internal Medicine and Oncology, Semmelweis University, Budapest, Hungary; ^6^ Semmelweis University Pediatric Center Tűzoltó Street Unit, Budapest, Hungary; ^7^ Department of Clinical Neurosciences, University of Cambridge, Cambridge, United Kingdom; ^8^ National Institute of Environmental Health, Department of Molecular Genetics and Diagnostics, Istenhegyi Genetic Diagnostic Centre, Budapest, Hungary; ^9^ Friedrich-Baur-Institute at the Department of Neurology, University Hospital, Munich, Germany; ^10^ Children’s Hospital of Eastern Ontario Research Institute, Division of Neurology, Department of Medicine, The Ottawa Hospital, and Brain and Mind Research Institute, University of Ottawa, Ottawa, ON, Canada; ^11^ Division of Infection and Immunity, School of Medicine, Cardiff University, Cardiff, United Kingdom; ^12^ McColl-Lockwood Laboratory for Muscular Dystrophy Research, Carolinas Medical Center, Charlotte, United States

**Keywords:** fukutin-related protein, muscular dystrophy, protein misfolding, missense mutation, chaperone, structural modelling

## Abstract

Fukutin-related protein (FKRP, MIM ID 606596) variants cause a range of muscular dystrophies associated with hypo-glycosylation of the matrix receptor, α-dystroglycan. These disorders are almost exclusively caused by homozygous or compound heterozygous missense variants in the *FKRP* gene that encodes a ribitol phosphotransferase. To understand how seemingly diverse *FKRP* missense mutations may contribute to disease, we examined the synthesis, intracellular dynamics, and structural consequences of a panel of missense mutations that encompass the disease spectrum. Under non-reducing electrophoresis conditions, wild type FKRP appears to be monomeric whereas disease-causing FKRP mutants migrate as high molecular weight, disulfide-bonded aggregates. These results were recapitulated using cysteine-scanning mutagenesis suggesting that abnormal disulfide bonding may perturb FKRP folding. Using fluorescence recovery after photobleaching, we found that the intracellular mobility of most FKRP mutants in ATP-depleted cells is dramatically reduced but can, in most cases, be rescued with reducing agents. Mass spectrometry showed that wild type and mutant FKRP differentially associate with several endoplasmic reticulum (ER)-resident chaperones. Finally, structural modelling revealed that disease-associated FKRP missense variants affected the local environment of the protein in small but significant ways. These data demonstrate that protein misfolding contributes to the molecular pathophysiology of FKRP-deficient muscular dystrophies and suggest that molecules that rescue this folding defect could be used to treat these disorders.

## 1 Introduction

Mutations in the gene encoding fukutin-related protein (FKRP) cause a spectrum of disorders ranging in severity from limb girdle muscular dystrophy type R9 (LGMDR9, previously known as LGMD2I) to congenital muscular dystrophy (CMD) with structural brain abnormalities resembling Walker-Warburg Syndrome (WWS) and Muscle-Eye-Brain (MEB) disease (Brockington et al., 2001a; Brockington et al., 2001b; Mercuri et al., 2003; Topaloglu et al., 2003; Beltran-Valero de Bernabe et al., 2004; Louhichi et al., 2004; Straub et al., 2018). *FKRP* mutations and mutations in its paralogue *FKTN* (encoding fukutin) are associated with hypo-glycosylation of the matrix receptor α-dystroglycan; a component of the dystrophin-glycoprotein complex (Yoshida-Moriguchi and Campbell, 2015; Manya and Endo, 2017). FKRP and fukutin are Golgi-localized enzymes that catalyze the transfer of ribitol 5-phosphate to the phospho-core M3 structure of α-dystroglycan using CDP-ribitol as a donor (Kanagawa et al., 2016). The combined enzymatic activity of FKRP, fukutin and a host of other enzymes creates an elaborate O-linked polysaccharide known as matriglycan on α-dystroglycan forming the binding site for several extracellular matrix proteins including laminin and neurexin (Yoshida-Moriguchi and Campbell, 2015).

FKRP can be divided into three domains; the N-terminal signal anchor (amino acids 1–44), the stem domain (amino acids 45–287) and the catalytic domain (288–495). The recently determined crystal structure of FKRP (amino acids 45–495) lacking the signal anchor sequence shows that the protein forms a tetramer composed of two protomeric dimers (Kuwabara et al., 2020). Each FKRP monomer contains a single disulfide bond (S–S) and a Zn^2+^ finger loop where four cysteine residues (C289, C296, C317, and C318) coordinate the divalent cation. The last cysteine residue C375 is buried within the protein. The protodimeric structure has been shown to be essential for enzymatic function where ribitol 5-phosphate binds to the catalytic domain of one subunit while the phosphate group on O-mannose is recognized by the stem domain on the adjacent subunit (Kuwabara et al., 2020). On the cytoplasmic face of the membrane, an intermolecular disulfide bond at C6 formed between adjacent molecules has been proposed to be involved in FKRP dimerization (Alhamidi et al., 2011).

Numerous pathogenic *FKRP* variants have been described in muscular dystrophy patients (https://databases.lovd.nl/shared/genes/FKRP). Most of these variants are missense mutations that are distributed along the entire coding sequence which is encoded by a single exon (Brockington et al., 2001a; Ortiz-Cordero et al., 2021a). Homozygous mutations tend to result in a milder disease severity than compound heterozygous mutations (Johnson and Statland, 2022). While mutations located in the catalytic domain of FKRP appear to be associated with the most severe phenotypes, there is no clear correlation between the location of these mutations, the substituted amino acid and disease severity (Ortiz-Cordero et al., 2021a). The mildest and most common form of muscular dystrophy involving *FKRP*, LGMDR9, is frequently associated with the mutation, c.826C>A (p.L276I) (Brockington et al., 2001b). LGMDR9 can be caused by homozygous p.L276I mutations, compound heterozygous missense mutations involving p.L276I and another missense mutation or frameshifting loss of function mutations (Stensland et al., 2011).

Previous work in our laboratory has shown that endoplasmic reticulum (ER)-retention and accelerated proteasomal degradation of FKRP mutants may account, at least in part, for the reduced α-dystroglycan glycosylation in patients (Esapa et al., 2002; Esapa et al., 2005). Proteins in the secretory pathway that are trafficked to or through the Golgi apparatus, are scrutinized by an elaborate quality control mechanism involving the concerted actions of a series of molecular chaperones that is initiated in the ER (Ellgaard and Helenius, 2003; Marinko et al., 2019). Many genetic diseases such as cystic fibrosis are associated with protein misfolding (Valastyan and Lindquist, 2014). In these disorders, missense mutations, deletions, or insertions impair folding of the mutant protein often resulting in its ER-associated degradation (ERAD). Importantly, some of these variants, including the common ΔF508 mutation in the cystic fibrosis transmembrane conductance regulator (CFTR:c.1521_1523del (p.Phe508del)), may retain functionality (Drumm et al., 1991) and can be rescued by pharmacological intervention (Gamez et al., 2018). Thus, stringent quality control can result in degradation or a failure to deliver functional proteins to their appropriate cellular destination resulting in disease (Valastyan and Lindquist, 2014).

Predicated on our original study that showed some FKRP mutations produce proteins that are ERAD substrates, here we examine the impact of a panel of disease-associated FKRP mutations on the synthesis and folding of the mutated protein using a range of biochemical, imaging, and molecular modelling techniques. We show that most disease-associated FKRP mutations adopt non-native conformations indicative of misfolded proteins.

## 2 Results

### 2.1 Most FKRP mutations result in protein misfolding

**FIGURE 1:**
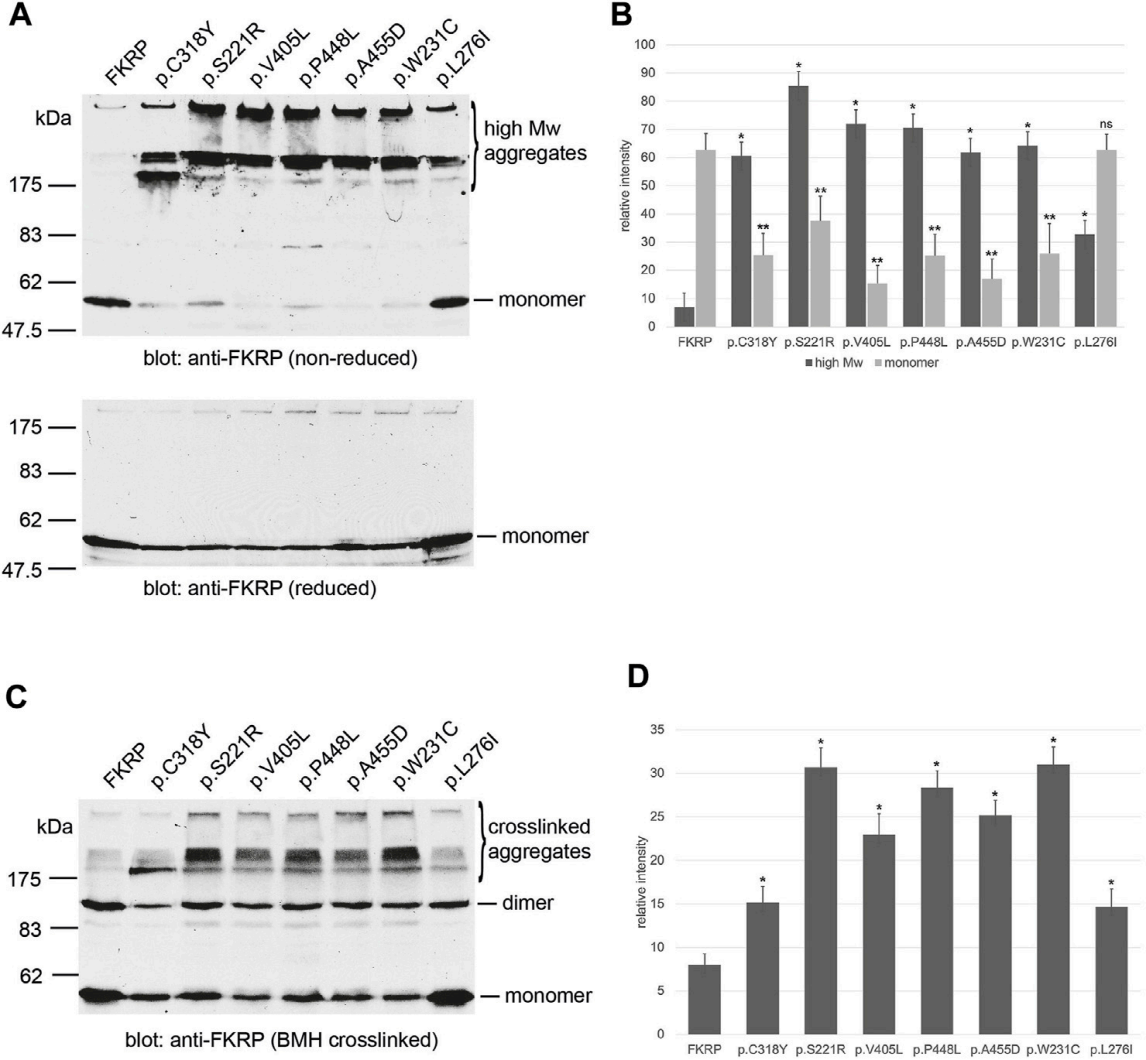
Biochemical analysis of FKRP synthesis in heterologous cells. Detection of wild type and mutant FKRPs by non-reducing PAGE **(A)**. Proteins prepared from transfected COS-7 cell extracts were denatured in the absence (non-reducing) or presence (reduced) of DTT before electrophoresis. Western blots were probed with the anti-FKRP antibody. All the FKRP mutants migrated as high molecular weight aggregates under non-reducing electrophoresis conditions These high molecular weight aggregates were abolished by the addition of DTT. By contrast, most of the wild type FKRP migrated as a monomer under non-reducing electrophoresis conditions. p.L276I gave an intermediate pattern and was detected as a folded monomer and in aggregates under non-reducing conditions. Quantitation of FKRP levels in DSP-treated cells **(B)**. Relative levels of high molecular weight (>175 kDa) and monomeric (57 kDa) protein were determined for each construct. Values are displayed as mean relative intensity ± standard deviation. Student’s t-test were used for pairwise comparisons of the means between FKRP and each variant (* = *p* < 0.01 for high molecular weight aggregates; **p < 0.01 for FKRP monomers; ns, not significant). BMH-crosslinking of wild type and mutant FKRPs **(C)**. Proteins were prepared from COS-7 cells transfected with various FKRP constructs that were lysed in the presence of BMH. Proteins were resolved under denaturing conditions, blotted and probed with the anti-FKRP antibody. Wild type FKRP is resolved as a monomer and BMH-crosslinked dimer in mammalian cells. The majority of the FKRP mutants formed high molecular weight BMH-crosslinked products in addition to the dimeric and monomeric forms of the protein. Levels of the crosslinked dimer and monomer appear reduced relative to wild type FKRP in all cases. Quantitation of FKRP levels in BMH-treated cells **(D)**. Levels of high molecular weight (>175 kDa) crosslinked aggregates were determined for each construct. Values are displayed as mean relative intensity ± standard deviation. Student’s t-test were used for pairwise comparisons of the means between FKRP and each variant (* = *p* < 0.01 for high molecular weight aggregates).

To investigate how the spectrum of FKRP mutations may alter protein synthesis and folding, we examined the steady state levels of FKRP, and a panel of disease-associated mutants (Table 1) using non-reducing polyacrylamide gel electrophoresis (PAGE) and *in vivo* crosslinking. In addition to the previously described variants, a homozygous p.W231C (c.693G>C) substitution was found in a patient with severe LGMDR9 (personal communication, Prof. Hanns Lochmüller). A heterozygous p.W231C variant has also been described alongside the c.1433T>C (p.I478T) variant in a patient with MEB (Mercuri et al., 2009). p.W231C is predicted to be pathogenic and has a REVEL score of 0.749 (Table 1). Similar results (AlphaMissense score of 0.99, pathogenic) were obtained using artificial intelligence-based pathogenicity predictions extracted from the AlphaMissense database (Cheng et al., 2023). The only significant difference between the REVEL and AlphaMissense predictions are for p.L276I that AlphaMissense erroneously classifies as benign. Proteins were resolved by PAGE with and without (non-reducing) pre-treatment with the reducing agent dithiothreitol (DTT) as described previously (Esapa et al., 2007). In each case the synthesis of the mutant protein was compared alongside wild type FKRP. Under non-reducing electrophoresis conditions, wild type FKRP migrates as a monomer of approximately 57 kDa with trace amounts present in higher molecular weight regions of the gel (Figure 1A). By contrast, all the FKRP mutants formed high molecular weight aggregates (>175 kDa) and produce, except for p.L276I, only small amounts of the folded monomer in the absence of the reducing agent (Figure 1A). At the extremes of disease severity, p.C318Y was resolved as two major high molecular weight products with trace levels of monomer while p.L276I was observed as a 57 kDa monomer but also formed high molecular weight species (>175 kDa) when compared to wild type FKRP (Figure 1A). Ratiometric western blot quantitation showed that the levels of FKRP in high molecular weight aggregates (>175 kDa) were significantly increased for all disease-causing FKRP variants when compared to wild type FKRP (Figure 1B). Similarly, with the exception of p.L276I, levels of the 57 kDa monomer were significantly reduced for all variants compared to the wild type protein (Figure 1B). Treatment of the cell extracts with reducing agents prior to electrophoresis removed the high molecular weight products allowing FKRP and each variant to be resolved as a 57 kDa protein (Figure 1A, lower panel).

**TABLE 1:** *FKRP* patient mutations modelled in this study.

Mutation	Disease association	Allele frequency	REVEL scores	AlphaMissense scores	Localization
FKRP	na	na	na	na	Golgi
p.S221R	CMD + CNS	nr	0.747	0.988	ER
p.W231C	LGMDR9	nr	0.749	0.990	ER/weak Golgi
p.L276I	LGMDR9	1.1e-3	0.700	0.292	Golgi/weak ER
p.C318Y	WWS-like	nr	0.937	0.997	Golgi/ER/some aggregates
p.V405L	CMD + CNS	nr	0.878	0.882	ER
p.P448L	CMD	8.97e-6	0.916	0.897	ER/weak Golgi
p.A455D	CMD + CNS	nr	0.819	0.963	ER

For comparative purposes, the REVEL (Rare Exome Variant Ensemble Learner) and AlphaMissense scores for each *FKRP* mutation were determined (Ioannidis et al., 2016; Cheng et al., 2023). Allele frequencies were extracted from the Genome Aggregation Database (gnomAD v2.2.1 database) (https://gnomad.broadinstitute.org/about) (Chen et al., 2022). Primary citation for each variant is as follows; FKRP, p.P448L (Brockington et al., 2001a); p.S221R (Topaloglu et al., 2003); p.W231C, this study, p.L276I, (Brockington et al., 2001b); p.C318Y, (Beltran-Valero de Bernabe et al., 2004); p.V405L, p.A455D, (Louhichi et al., 2004). Na, not applicable; nr, not reported.

**FIGURE 2:**
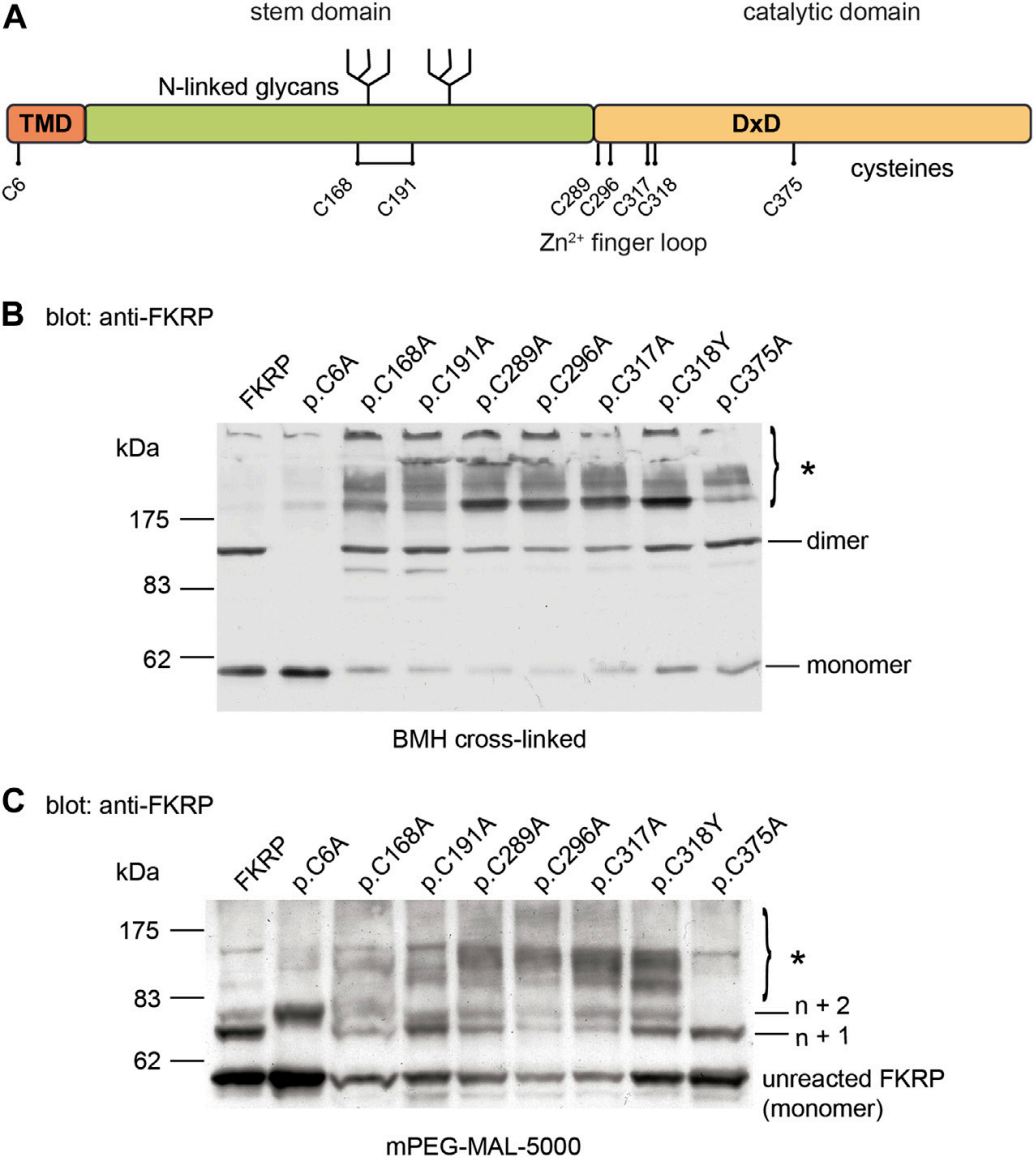
Cysteine mutations affect FKRP biosynthesis. Schematic showing the position of the cysteines on FKRP **(A)**. The location of the two N-linked glycosylation sites, the DxD motif and the transmembrane domain (TMD) are also shown. Cell extracts prepared from HEK293T cells transfected with the indicated FKRP constructs were crosslinked with BMH **(B)** or PEGylated with the alkylating agent mPEG-MAL-5000 **(C)**. Crosslinked and PEGylated proteins were resolved by SDS-PAGE and detected with the anti-FKRP antibody. Asterisks mark PEGylated and crosslinked proteins (see text for details).

Several Golgi-resident glycosyltransferases including FKRP are known to contain intra- and inter-molecular disulfide bonds that are essential for their enzymatic activity (Qian et al., 2001; Kuwabara et al., 2020). To test the hypothesis that FKRP missense mutations may cause misfolding through an inability to form legitimate disulfide bonds, we prepared lysates from transfected cells in the presence of the thiol reactive crosslinker, bismaleimidohexane (BMH). BMH is a homobifunctional reagent with a 1.3 nm spacer that forms non-hydrolyzable bonds between exposed and accessible free thiol groups. Thus, the crosslinker can form intramolecular bonds between free thiols and intermolecular bonds between adjacent FKRP molecules or between FKRP and associated proteins such as chaperones. BMH-crosslinked FKRPs were resolved by denaturing PAGE in the presence of 20 mM DTT to reduce inter- and intramolecular disulfide bonds formed by unreacted proteins as described previously (Esapa et al., 2007). Western blot analysis using the anti-FKRP antibody showed that FKRP could be resolved into two distinct bands that correspond to the FKRP monomer and putative dimer (Figure 1C; Figure 2). In addition to the monomer and putative dimer, each mutant also formed high molecular weight BMH-crosslinked aggregates (Figure 1C) that were less apparent for wild type FKRP. Blot quantification revealed that each variant produced significantly higher levels of high molecular weight BMH-crosslinked material compared to FKRP (Figure 1D). Similar results were observed for a range of mutations (eg. p.R54W, p.Y182C and p.R352G) that cause LGMDR9 demonstrating that *FKRP* mutations associated with milder phenotypes still form high molecular weight, BMH-crosslinked aggregates (data not shown). Taken together, these data suggest that most FKRP mutants have an impaired ability to adopt a native conformation due to a defect in the formation of intramolecular disulfide bonds.

We also examined the subcellular localization of each mutant in transfected C2C12 myotubes (Supplementary Figure S1). As described previously, several FKRP mutants including p.W231C (see below) appeared to be retained in the ER (Esapa et al., 2005). However, some variants such as p.C318Y were clearly trafficked to the Golgi apparatus even though they have the biochemical properties indicative of misfolded proteins.

### 2.2 Effect of cysteine substitutions on mixed disulfide formation

Protein misfolding is both a cause and consequence of aberrant disulfide bond formation (Kosuri et al., 2012). To investigate the role of intra- and/or intermolecular disulfide bond formation on FKRP synthesis, we used cysteine-scanning mutagenesis to create a panel of mutants where each of the eight cysteine residues was mutated to alanine. Based upon the predicted topology of FKRP as a type II membrane protein, human FKRP has eight cysteine residues, only one of which is located on the cytoplasmic face of the Golgi apparatus membrane (Figure 2A). The crystal structure of FKRP shows that each monomer contains a single disulfide bond formed by C168 and C191 and a zinc finger loop formed by C289, C296, C317, and C318, therefore cysteine mutants should interfere with intramolecular interactions potentially resulting in misfolding of the mutant protein (Kuwabara et al., 2020). The WWS mutation, p.C318Y, was included in this series in place of the corresponding alanine mutant. HEK293T cells transfected with each of the cysteine mutants were treated with BMH as described above. As before, wild type FKRP was resolved as two major bands: a 57 kDa band corresponding to the FKRP monomer and 110 kDa protein corresponding to the FKRP dimer (Figure 1C; Figure 2B). The p.C6A mutation, located on the cytoplasmic face of the membrane produced a single product corresponding to the FKRP monomer (Figure 2B). These data suggest that the homodimer formed by BMH crosslinking is likely to occur between free thiol groups on adjacent FKRP molecules at the cytoplasmic face of the ER or Golgi membrane and that endogenous FKRP, consistent with the crystal structure (Kuwabara et al., 2020), is not a disulfide-linked dimer as previously proposed (Alhamidi et al., 2011). Furthermore, BMH crosslinking suggests that individual FKRP molecules are in proximity since they react with BMH that has a 1.3 nm spacer. The p.C6A mutant does not form high molecular weight complexes with BMH and is trafficked to the Golgi apparatus suggesting that p.C6A does not cause FKRP to misfold. The other cysteine mutants yielded reduced levels of monomeric and dimeric FKRP in comparison to the wild-type protein (Figure 2B). In common with the disease-associated FKRP mutations, the luminal cysteine mutants formed the characteristic high molecular weight aggregates indicative of misfolded proteins. Interestingly, p.C375A was not crosslinked to the same extent as the other mutants, although its behavior differed from wild type FKRP (see below).

Covalent modification of proteins with the thiol-reactive polymer mPEG-MAL-5000 (PEGylation) has been used to determine disulfide bond formation in a range of membrane proteins (Guo et al., 2005; Chen et al., 2016; Braakman et al., 2017). In addition to cysteine mutagenesis, we adapted the use of mPEG-MAL-5000 (which reacts with reduced thiols) as a probe to determine the reactivity of reduced cysteine residues in FKRP. In these experiments, proteins are covalently modified with mPEG-MAL-5000, adding approximately 10 kDa to the size of the polypeptide per reactive thiol. Detergent extracts were prepared from HEK293T cells transfected with different FKRP constructs and treated with mPEG-MAL-5000 under native conditions. mPEG-MAL-5000 reacted with wild type FKRP, yielding a major PEGylated product of approximately 67kDa, corresponding to the addition of one mPEG molecule (n + 1) and a minor product equivalent to n + 2 (Figure 2C). A 57 kDa band corresponding to unreacted FKRP was also detected after PEGylation (Figure 2C). These data suggest that FKRP has at least two free thiols that can be alkylated in the native protein.

Having established that mPEG reacts with FKRP, we compared the PEGylation profile of wild type FKRP with the panel of cysteine mutants described above. The mutation p.C6A resulted in the loss of the first PEGylated product (n + 1, Figure 2C) and the appearance of second product corresponding to addition of three mPEG-MAL-5000 molecules to FKRP (n + 2, Figure 2C). This result suggests that luminal thiols may become accessible in this mutant suggesting that cytosolic determinants may also influence the folding of FKRP. There also appeared to be an increase in the levels of the non-alkylated p.C6A compared to wild type FKRP (Figure 2C). The p.C375A mutant reacted with two mPEG-MAL-5000 molecules that looked almost identical to wild type FKRP (n + 1, Figure 2C). These data are consistent with C375 being buried within the catalytic domain of FKRP where it is unpaired and does not have access to the solvent void (Kuwabara et al., 2020). The other six mutants produced complex patterns of PEGylated products indicating the presence of multiple additional free thiols (asterisk, Figure 2C). Each of these mutants (p.C168A, p.C191A, p.C289A, p.C296A, p.C317A, and p.C318Y) had higher levels of PEGylated FKRP compared to the wild type protein. The appearance of diffuse higher molecular weight PEGylated products suggests that each of these mutants have additional, accessible thiol groups that are unreactive in the wild type protein (Figure 2C). Collectively, these data suggest that most cysteine substitutions cause FKRP to misfold by disrupting the formation of intramolecular disulfide bonds (C168-C191) or the zinc finger loop (C289, C296, C317, and C318).

### 2.3 Intracellular dynamics of FKRP mutants in ER membranes

**FIGURE 3:**
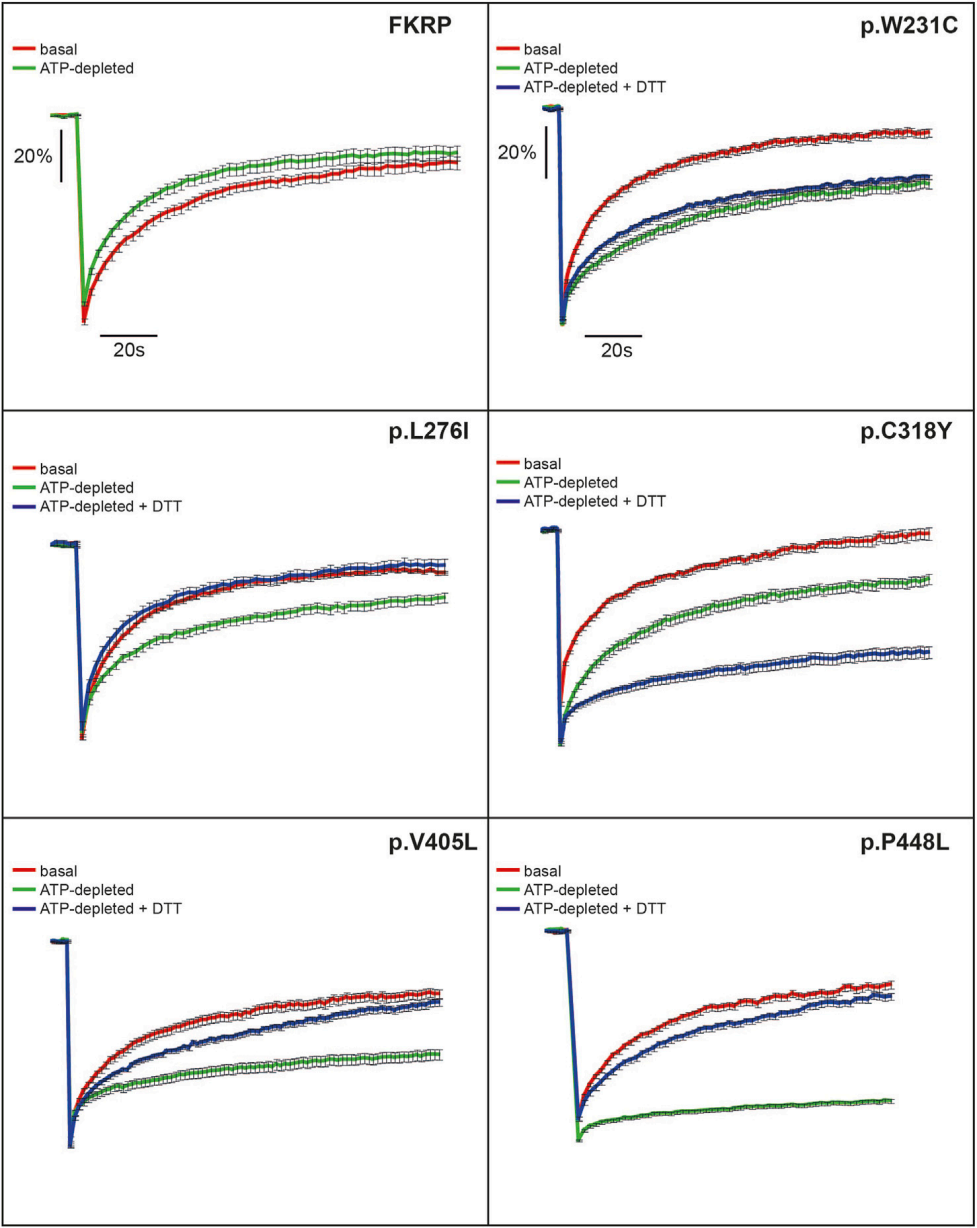
FRAP analysis of FKRP dynamics in live cells. EYFP-tagged wild type FKRP and the mutants, p.W231C, p.L276I, p.C318Y, p.V405L and p.P448L were subjected to FRAP analysis to determine mobility parameters in the ER after treatment with brefeldin A. In each panel, the red line shows fluorescence recovery (%) in D-glucose-containing basal media. Fluorescence recovery in ATP-depleted media is shown in green, whilst fluorescence recovery in ATP-depleted media containing 5 mM DTT is shown in blue. Each point represents the mean fluorescence recovery from analysis of at least 10 cells ± SEM (standard error of the mean). Similar results were obtained in three separate experiments. Fluorescence recovery (scale bar = 20%) is shown on the vertical axis whereas time (scale bar = 20s) is depicted on the horizontal axis.

In addition to the biochemical techniques described above, we used fluorescence recovery after photobleaching (FRAP) in live cells to determine the intracellular mobility of FKRP and mutants thereof. It has been previously shown that several misfolded proteins including a temperature sensitive mutant of vesicular stomatitis virus G-protein (VSV-G) and the common p.Phe508del CFTR (cystic fibrosis transmembrane conductance regulator) variant have impaired intracellular mobility in ATP-depleted cells (Nehls et al., 2000; Haggie et al., 2002). To determine whether disease-associated FKRP variants behaved like typical misfolded proteins in live cells, we compared the diffusion coefficient (*D*) and mobile fraction (*M*_*f*_, the percentage of molecules that can undergo diffusion in to the FRAP zone) of wild type FKRP and each variant in cells under basal conditions with ATP-depleted cells and with ATP-depleted cells supplemented with DTT (Figure 3). ATP-depletion leads to aberrant interchain disulfide bonding, crosslinking and aggregation of misfolded VSV-G with ER chaperones such as BiP/GRP78 (one of the chaperones associated with FKRP, see below) that can be reversed by reducing agents such as DTT (Gallione and Rose, 1985; Dorner et al., 1990; de Silva et al., 1993). Furthermore, reducing agents have been shown to dissociate calnexin from its substrates suggesting that calnexin also plays a role in disulfide bond formation (Tector and Salter, 1995). Thus, the aggregation of chaperones and clients in ATP-depleted cells affects the intracellular mobility of misfolded proteins in the ER that can be examined using FRAP (Nehls et al., 2000; Haggie et al., 2002). EYFP-tagged FKRP constructs were transfected into COS-7 cells and a high intensity focal laser beam was used to photobleach an area of the ER. Addition of EYFP to the C-terminus of FKRP did not appear to affect the subcellular localization of the wild type protein or the different variants (Supplementary Figure S1) (Esapa et al., 2005). Following photobleaching we monitored the fluorescence recovery of the various proteins in the presence and absence of ATP. In each experiment, at least 10 cells were imaged, and each experiment was performed a minimum of three times. The diffusion coefficient for wild type FKRP were comparable to those described for membrane proteins in the ER; *D* = 0.54 ± 0.12  μm^2^ s^−1^ compared with 0.49 ± 0.06  μm^2^ s^−1^ for VSV-G protein (Nehls et al., 2000). We also calculated the *M*_*f*_ for each protein under different conditions. The *M*_*f*_ of wild type FKRP was similar under basal and ATP-depleted conditions (Table 2; Figure 3).

**TABLE 2:** Effects of ATP depletion and reducing conditions on the diffusion coefficients and mobile fraction values for FKRP-EYFP chimeras and a panel of mutants in COS-7 cells.

Construct	*D* (µm^2^ s^-1^)	*M*_*f*_ (%)	n
**FKRP**	0.54 ± 0.12	77.0 ± 2.7	10
**FKRP - ATP**	0.64 ± 0.05	80.0 ± 1.2 (3.4e-3)	11
**p.W231C**	0.55 ± 0.06	88.5 ± 5.0	10
**p.W231C - ATP**	0.20 ± 0.08 (1.8e-9)	64.5 ± 4.0 (6.2e-10)	10
**p.W231C - ATP + DTT**	0.33 ± 0.04 (1.6e-8)	67.2 ± 3.5 (1.9e-9)	10
**p.L276I**	0.32 ± 0.07	73.0 ± 6.3	10
**p.L276I - ATP**	0.24 ± 0.04 (5.7e-3)	75.0 ± 12.0	10
**p.L276I - ATP + DTT**	0.38 ± 0.04	76.0 ± 10.0	10
**p.C318Y**	0.84 ± 0.11	91.0 ± 3.6	11
**p.C318Y - ATP**	0.30 ± 0.06 (5.8e-12)	72.8 ± 3.2 (6.3e-11)	11
**p.C318Y - ATP + DTT**	0.19 ± 0.03 (3.2e-14)	55.9 ± 6.5 (1.1e-12)	11
**p.V405L**	0.48 ± 0.08	74.1 ± 4.0	10
**p.V405L - ATP**	0.36 ± 0.09 (5.0e-3)	42.7 ± 5.2 (3.5e-12)	11
**p.V405L - ATP + DTT**	0.25 ± 0.05 (7.7e-7)	70.1 ± 3.5	10
**p.P448L**	0.36 ± 0.05	78.0 ± 2.3	10
**p.P448L - ATP**	0.30 ± 0.10	27.0 ± 2.1 (<2.2e-16)	10
**p.P448L - ATP + DTT**	0.33 ± 0.08	76.0 ± 5.2	10

Diffusion coefficients (*D* ± standard deviation) and mobile fractions (*M*_*f*_ ± standard deviation) of FKRP-EYFP, and variants under different experimental conditions. Statistical analyses of *D* and *M*_*f*_ values for each variant under different conditions were performed using a two-tailed Student’s t-test. The table lists *p* values (≤0.01) for pairwise comparisons between each construct under basal conditions and in ATP-depleted (-ATP) cells or under basal conditions and in ATP-depleted supplemented with DTT (- ATP + DTT). Each experiment was performed at least three times.

Next, we examined the intracellular dynamics of a range of FKRP mutants under the same conditions. By contrast to FKRP, ATP-depletion resulted in statistically significantly reductions in *D* for each variant (Table 2). Except for p.L276I, the change in *D* was accompanied by significant reductions in *M*_*f*_ for each protein indicative of protein misfolding (Nehls et al., 2000; Haggie et al., 2002). Interestingly, DTT was able to reverse the effects of ATP-depletion on *D* and *M*_*f*_ for p.L276I, p.V405L and p.P448L but not for the variants containing cysteine substitutions. p.L276I behaved similarly to wild type FKRP in the presence of ATP (Table 2; Figure 3). However, following ATP-depletion, p.L276I showed a slight slowing in its diffusion coefficient that was reversed by DTT (Figure 3). By contrast, p.P448L showed a severe reduction in *M*_*f*_ following ATP depletion that was almost completely reversed by DTT treatment (Figure 3; Table 2). It is noteworthy that the behavior of p.P448L in these experiments was very similar to the behavior of misfolded VSV-G under the same conditions (Nehls et al., 2000). Similar results were obtained for wild type FKRP, p.L276I, and p.P448L in C2C12 myoblasts (Supplementary Figure S2).

The remaining mutants, p.W231C, p.C318Y, and p.V405L each exhibited markedly different fluorescence recovery curves under basal and ATP-depleted conditions (Figure 3). Under basal conditions p.W231C behaved comparably to p.L276I however, by contrast to p.L276I and p.P448L, DTT treatment did not increase *D* or *M*_*f*_ of p.W231C (Figure 3; Table 2). p.V405L also showed a significant reduction in *D* and *M*_*f*_ following ATP-depletion (Figure 3; Table 2). In common with the other mutants, DTT treatment gradually increased *M*_*f*_ of p.V405L after ATP-depletion but with a lower *D*. Finally, the p.C318Y mutant was found to be highly mobile in the ER of untreated COS-7 cells (*D* = 0.84 ± 0.11 Table 2). Although, ATP-depletion reduced both *D* and *M*_*f*_ of p.C318Y (as described for the other mutants), DTT treatment unexpectedly lowered each of these parameters, further exacerbating the effect of ATP-depletion (Figure 3; Table 2). These data show that all disease-associated FKRP mutants behave like misfolded proteins and highlight idiosyncratic differences in the dynamic properties of the variants p.W231C and p.C318Y that involve cysteine residues.

### 2.4 Reducing agents may improve trafficking and steady state levels of mutant FKRPs

**FIGURE 4:**
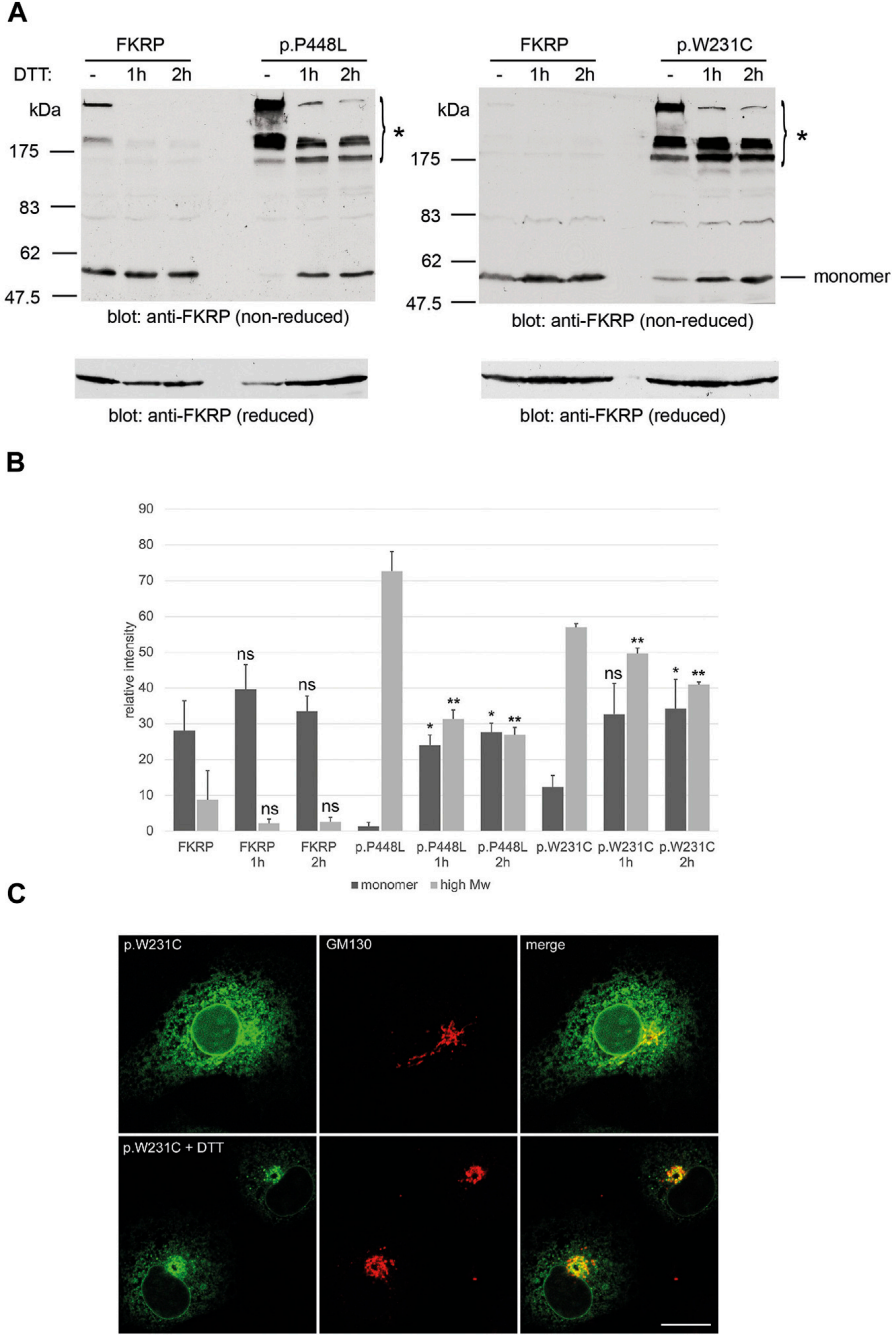
Effects of DTT on FKRP synthesis and trafficking. DTT treatment improves folding of FKRP mutants, p.P448L and p.W231C **(A)**. Twenty-4 hours after transfection with the relevant expression construct, HEK293T cells were incubated at 37°C with 5 mM DTT in complete growth medium for the indicated times. Cells extracts were processed for non-reducing SDS-PAGE and FKRP was detected by immunoblotting with the anti-FKRP polyclonal antibody. For each mutant, DTT treatment increases the level of folded FKRP with concomitant reduction in the amount of high molecular weight aggregated protein (asterisk). Wild type FKRP has been included in both experiments to allow direct comparison. Reduction of each sample with 2-mercaptoethanol (lower panels in **(A)**) disrupts the high molecular weight aggregates allowing FKRP to be resolved as single 57 kDa protein monomer. Quantitation of FKRP levels in DTT-treated cells **(B)**. The relative levels of high molecular weight (>175 kDa) and monomeric (57 kDa) protein were determined for FKRP, p.W231C and p.P448L after living cells were incubated with DTT for 1 or 2 h. Values are displayed as mean relative intensity ± standard deviation. Student’s t-test were used for pairwise comparisons of the means between each construct in untreated and DTT treated cells (* = *p* < 0.01 for high molecular weight aggregates; **p < 0.01 for FKRP monomers; ns, not significant). DTT treatment may improve ER to Golgi trafficking of p.W231C **(C)**. Two hours after transfection COS-7 cells transfected with the p.W231C cDNA were treated with 0.5 mM DTT and incubated for a further 16 h. Cells were stained with anti-FKRP (green) and anti-GM130 (red) antibodies. In DTT-treated cells, p.W231C is concentrated at the Golgi apparatus whilst levels in the ER are reduced. Scale bar = 20 μm.

Having found that misfolded FKRP mutants participate in complex disulfide bonded interactions, which in some cases may result in ER-retention of the mutant protein, we hypothesized that if these bonds could be disrupted, “mobile” FKRP mutants may traffic from the ER to the Golgi apparatus. For these studies we compared the behavior of wild type FKRP with the well-studied p.P448L variant and p.W231C, that has the potential to interfere with physiological disulfide bond formation. Living COS-7 cells transfected with the mutants p.P448L and p.W231C were incubated with 5 mM DTT for 1 h or 2 h prior to processing for non-reducing PAGE and immunocytochemistry (Figure 4A). DTT treatment of p.P448L and p.W231C for 1 or 2 h improved the yield of the FKRP monomer while reducing the levels of high molecular weight aggregates that were originally formed (Figure 4A). Extracts from cells expressing wild type FKRP showed no obvious increase in the formation of the FKRP monomer (Figure 4A). Western blot quantitation showed that the levels of p.P448L and p.L276I in high molecular weight aggregates (>175 kDa) were significantly reduced after 1 and 2 h incubation with DTT (Figure 4B). By contrast, the relatively low levels of FKRP in high molecular weight aggregates were not significantly reduced by DTT treatment. Conversely, DTT treatment for 2 h significantly increased the relative levels of monomeric protein (57 kDa) for the p.P448L and p.L276I variants but had no apparent effect on wild type FKRP (Figure 4B). Finally, confocal imaging of cells expressing p.W231C following DTT treatment, showed an apparent increase in p.W231C immunoreactivity at the Golgi apparatus (labeled with GM130) and a reduction of labelling intensity in the ER (Figure 4C). These data suggest that DTT treatment reduces the aggregation of certain misfolded FKRP variants and may improve the trafficking of p.W231C in living cells. However, it is formally possible that DTT treatment may perturb the secretory pathway and/or enhance the ER clearance of p.W231C without directly affecting protein trafficking between the ER and Golgi apparatus.

### 2.5 FKRP mutants differentially associate with ER-resident chaperones

**FIGURE 5:**
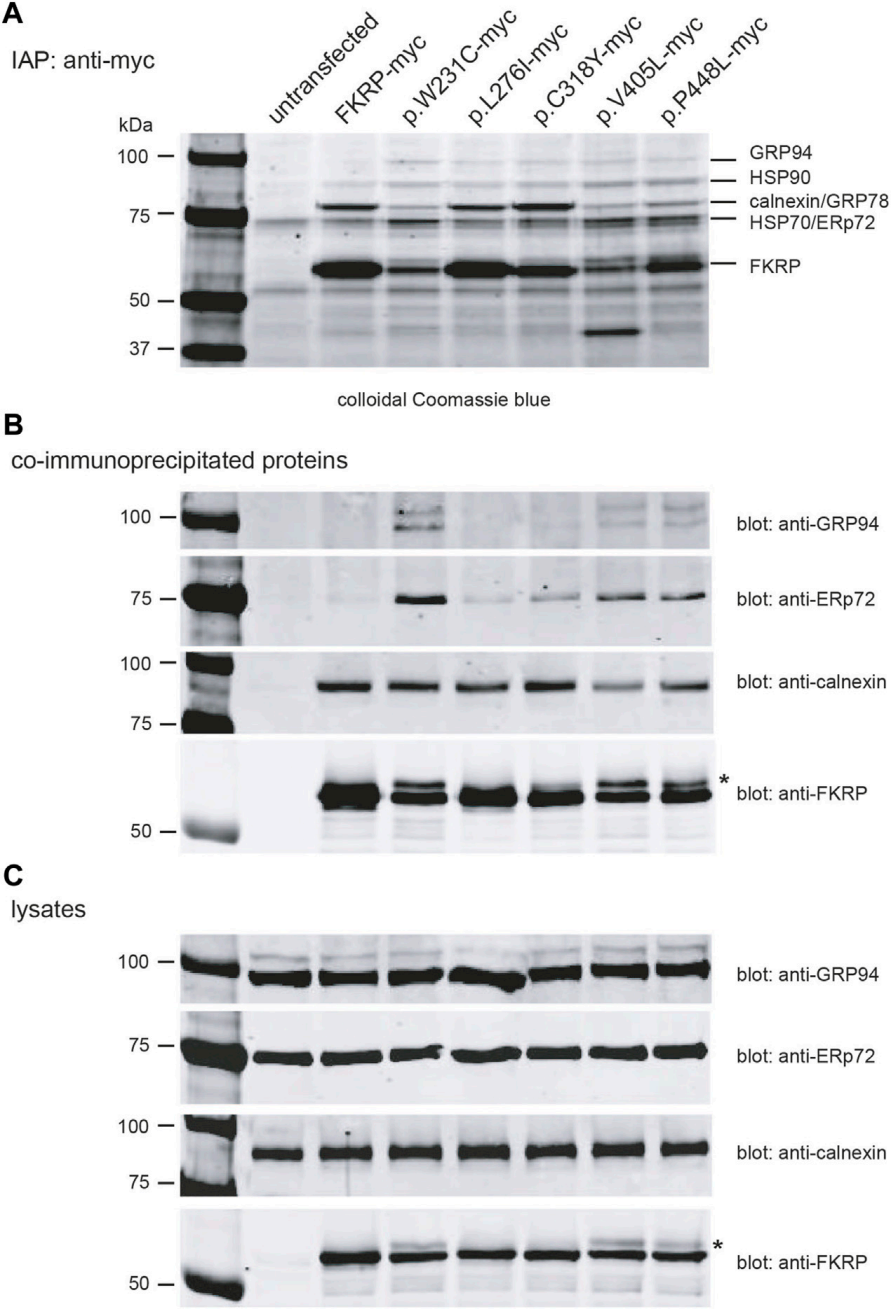
Identification of FKRP-associated chaperones using mass spectrometry. Live HEK293T cells transfected with the indicated constructs were incubated with the DSP prior to lysis. Cells were lysed in RIPA buffer and cleared lysates used for immunoaffinity purification IAP using anti-myc 9E10 conjugated beads. Samples were resolved on a 4%–12% polyacrylamide gradient gel, stained with colloidal Coomassie blue and processed for mass spectrometry **(A)**. Western blotting immunoprecipitated proteins prepared from cell extracts (without DSP) with a range of antibodies confirmed the presence of FKRP, GRP94, ERp72 and calnexin **(B)**. For comparative purposes, the cell lysates from each transfection were also western blotted with anti-GRP94, anti-ERp72, anti-calnexin and anti-FKRP antibodies **(C)**. The asterisks in **(B,C)** highlight the higher molecular weight FKRP isoforms that are found for the mutants p.W231C, p.V405L and p.P448L. These isoforms possibly represent ER-retained misfolded protein where the two endogenous N-linked glycans on FKRP (N172 and N209) have not been processed by the ER glucosidases.

Having found that most disease-associated FKRP-mutations appear to result in misfolding of the mutant protein, we used Fourier transform ion cyclotron resonance mass spectrometry (FTICR-MS) to search for intracellular chaperones that interact with FKRP. HEK293T cells transfected with a panel of myc-tagged FKRP mutants were treated with the cell permeable crosslinker DSP to preserve low affinity interactions prior to lysis and immunoaffinity purification (IAP). Proteins were resolved by SDS-PAGE (Figure 5A), stained and individual bands were excised and processed for FTICR-MS. Visual inspection of the colloidal Coomassie blue stained gel showed marked differences in the levels of wild type and mutant FKRP immunoprecipitated from each transfection and qualitative differences in the profile of co-purifying proteins (Figure 5A). For example, the Coomassie stained gel appears to show that higher levels of the proteins that traffic to the Golgi apparatus (FKRP, p.L276I and p.C318Y) were recovered by IAP (Figure 5A). By contrast, the ER-retained mutants, p.W231C, p.V405L, and p.P448L appeared to be present at lower levels compared to wild type FKRP (Figures 5A,B). Furthermore, a higher molecular weight isoform was also apparent in cells expressing these mutants (asterisk in Figure 5B) and the cell lysates (Figure 5C). Since p.W231C, p.V405L and p.P448L are ER-retained proteins the higher molecular product may represent unprocessed folding intermediates or ERAD substrates where the N-linked glycans on FKRP (Esapa et al., 2005; Alhamidi et al., 2011) have not been trimmed by the ER-resident glucosidases (Wang and Kaufman, 2016).

FTICR-MS was used to identify the additional proteins co-purifying with FKRP (Table 3 and Supplementary Table S2). Several cytosolic and ER-resident chaperones including calnexin, that was previously shown to bind to FKRP (Esapa et al., 2005), GRP94, GRP78 (BiP), the protein disulfide isomerase ERp72 and VCP, that is mutated in inclusion body myopathy, were found to co-purify with FKRP (Figure 5B). Western blot analysis of the immunoprecipitates with a range of anti-chaperone antibodies showed co-purification of ERp72, calnexin and GRP94 with wild type and mutant FKRP confirming their association (Figure 5B). It is noteworthy that many of the ER-resident chaperones appear to associate preferentially with certain FKRP mutants. For example, ERp72 and GRP94 appear to co-purify at higher levels with p.W231C whereas higher levels of calnexin are associated with wild type FKRP, p.L276I and p.C318Y. Taken together, these data suggest that wild type and mutant FKRPs interact with a range of cellular chaperones that are likely to participate in the folding and trafficking of nascent FKRP in the early secretory pathway.

**TABLE 3:** Proteins immunoaffinity purifying with wild type and mutant FKRP identified by mass spectrometry.

Protein ID/*Official gene symbol*/alias	XC score	Peptide count	MW
**NP_003290.1**/*HSP90B1* (heat shock protein 90 beta family member 1)/GRP94	260.30	31	92,411.2
**NP_005337.1**/*HSPA1B* (heat shock protein family A (Hsp70) member 1B)/HSP70.2	68.25	32	69,982.2
**NP_005518.2**/*HSPA1L* (heat shock protein family A (Hsp70) member 1 like)/HPS70-1L	40.20	20	70,361.4
**NP_009057.1**/*VCP *(valosin containing protein)/p97	30.14	3	89,265.9
**NP_005339.2**/*HSP90AA1* (heat shock protein 90 alpha family class A member 1)/HSP90A	126.22	18	84,620.7
**NP_031381.2**/*HSP90AB1* (heat shock protein 90 alpha family class B member 1)/HSP90B	238.21	35	83,212.2
**NP_005337.1**/*HSPA1B* (heat shock protein family A (Hsp70) member 1B)/HSP70.2	60.22	14	69,982.2
**NP_005338.1**/*HSPA5* (heat shock protein family A (Hsp70) member 5)/BIP, GRP78	286.22	47	72,288.5
**NP_004125.3**/*HSPA9* (heat shock protein family A (Hsp70) member 9)/GRP75	204.25	30	73,634.8
**NP_068814.2**/*HSPA2* (heat shock protein family A (Hsp70) member 2)/HPS70-2	80.23	10	69,978.0
**NP_057376.1**/*TRAP1* (TNF receptor associated protein 1)/HSP75	58.17	8	79,960.7
**NP_694881.1**/*HSPA8* (heat shock protein family A (Hsp70) member 8)/HSC70	50.19	7	53,484.5
**NP_004902.1**/*PDIA4* (protein disulfide isomerase family A member 4)/ERp72	88.19	13	72,887.1
**NP_001737.1**/*CANX* (calnexin)/CNX	60.17	8	67,526.0

Mass spectrometry statistics for chaperones co-immunoaffinity purifying with wild type and mutant FKRP, from transfected HEK293T cells. Corresponding peptides are show in Supplementary Table S2.

### 2.6 Structural modelling of FKRP variants

**FIGURE 6:**
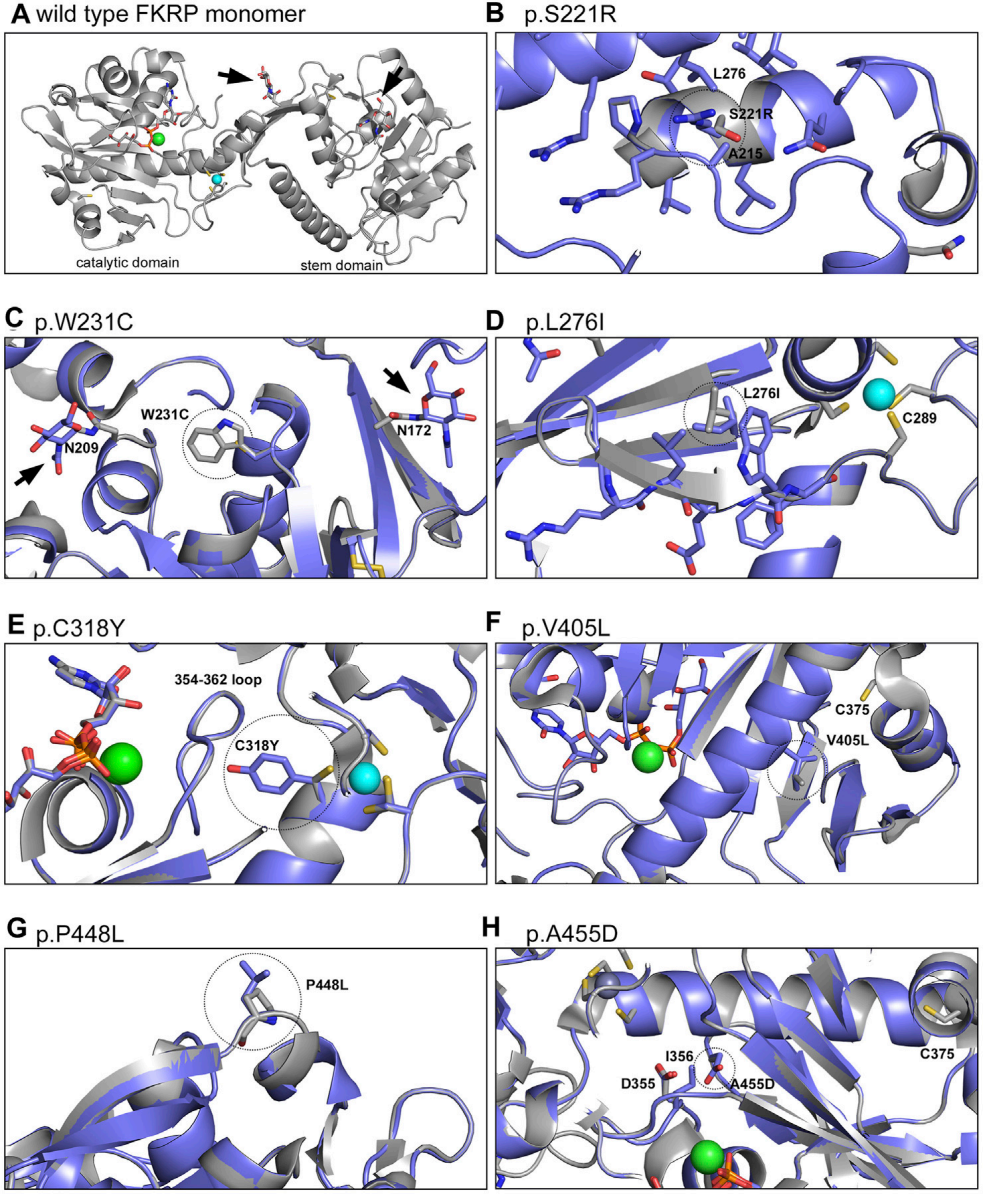
Structural models of FKRP missense mutations. The crystal structure of the wild type FKRP protein (6KAM, **(A)** was used to generate a range of models depicting the local environment of the following mutants: p.S221R **(B)**, p.W231C **(C)**, p.L276I **(D)**, p.C318Y **(E)**, p.V405L **(F)**, p.P448L **(G)** and p.A455D **(H)**. For most variants, the mutation changes the protein in small but significant ways potentially altering a range of intra- and intermolecular interactions that lead to misfolding. By contrast, p.P448L is located on a loop that extends into the solvent void and may create a hydrophobic patch that is required for the correct folding of FKRP. For each model the wild type protein is shaded gray while the variant (residue circled with dotted line) is represented in blue. The Mg^2+^/Ba^2+^ ion at the active site of FKRP is shown in green and the coordinated Zn^2+^ ion of the Zn^2+^ finger loop is shown in turquoise. Arrowheads denote the N-linked glycans at residues N172 and N209.

Finally, molecular dynamics simulations were used to provide insights into the predicted effect of different mutations on the structure of FKRP (Figure 6). Mutations associated with different disease severities (p.S221R, p.W231C, p.L276I, p.C318Y, p.V405L, p.A455D) introduced into the structure of FKRP (PDB:6KAM, Figure 6A) were found to modify the local environment of the protein in small but significant ways. p.S221R and p.L276I have previously been shown to alter the oligomerisation state of FKRP in solution and are associated with reduced enzymatic activity (Kuwabara et al., 2020). p.S221R is located at the intersubunit interface and could affect conformation of the loop immediately prior to the glycosylation site at N209, possibly interfering with the folding of the protein disrupting subunit assembly (Figure 6B). p.W231C may reduce the physical buffer between the two glycosylation sites at N172 and N209 leading to a small local structural perturbation (Figure 6C). p.W231C may also affect disulfide bonding leading to the formation of non-physiological oligomers as described above. p.L276I could destabilise the last strand of the beta sheet leading up to C289, one of the ligands of the important Zn^2+^ finger loop near the hinge between the stem and catalytic domains of FKRP (Figure 6D). C318 forms part of the Zn^2+^ finger loop of FKRP and the p.C318Y variant deprives the coordinate Zn^2+^ ion that stabilises the loop of one of its ligands, possibly leading to its abstraction from the site and the unfolding of the protein (Figure 6E) (Kuwabara et al., 2020; Ortiz-Cordero et al., 2021b). The side chain formed by the tyrosine substitution can only be accommodated by re-orientating the amino acid toward the 354–362 loop, pushing the loop closer to the Mg^2+^/Ba^2+^ ion found in the active site of FKRP. This rearrangement could either cause the exclusion of the cation or reshaping of the active site attenuating catalytic activity. Extra atoms in the side chain of the p.V405L substitution could lead to regional unfolding around the unpaired C375 that is normally buried in the core of the catalytic domain of wild type FKRP (Figure 6F). p.V405L could cause local unfolding around C375 allowing this unpaired cysteine residue to react with other cysteines in adjacent molecules leading to non-physiological oligomerisation. One other severe mutation, p.P448L, does not appear to have any obvious structural implications beyond exposing a hydrophobic patch to the solvent void (Figure 6G). P448 is located on a surface loop, away from any interfaces and has no direct impact on subunit interactions or formation of the active site. We speculate that p.P448L may result in local misfolding that affects binding to another protein (eg. fukutin or TMEM5) or a chaperone (Nishihara et al., 2018). Finally, the extra side chain atoms and acquired charge in the p.A455D substitution could push the loop around D355 and I356 into the binding site for the catalytic Mg^2+^/Ba^2+^ ion potentially altering the active site of FKRP (Figure 6H).

## 3 Discussion

Using a battery of complementary techniques, we show that *FKRP* mutations spanning a range of disease severities lead to misfolding of the mutated protein. Misfolding can affect protein function directly by altering its biological activity or indirectly, by impairing intracellular transport or assembly into large oligomeric complexes (Araki and Nagata, 2011). In the case of FKRP, misfolding could attenuate the enzymatic function of FKRP (Kanagawa et al., 2016), lead to degradation of the mutant protein by the ubiquitin proteasome system (Esapa et al., 2005) or impair trafficking in the early secretory pathway (Esapa et al., 2002; Henriques et al., 2019). While many *FKRP* variants impair trafficking, disease-associated missense mutations in residues 300–320 that form part of the Zn^2+^ finger loop of FKRP, can traffic to the Golgi apparatus but are functionally impaired (Supplementary Figure S1) (Henriques et al., 2019). This observation may partially explain the paradoxical finding that *Fkrp* mutant mice with the homozygous p.Y307N mutation are phenotypically normal while the same mutation in humans causes a severe congenital disorder (Beltran-Valero de Bernabe et al., 2004; Ackroyd et al., 2009; Brown et al., 2020). Over-expression of the p.P448L *Fkrp* variant can restore functional α-dystroglycan glycosylation and reduce muscle pathology in *Fkrp* mutant mice (Tucker et al., 2018). Similarly, Henriques and colleagues found that many mislocalised FKRP variants retain significant activity in assays for functional α-dystroglycan glycosylation (Henriques et al., 2019). Collectively, these data suggest that loss of enzymatic activity does not adequately explain the molecular pathology of the range of disorders caused by *FKRP* mutations.

In addition to N-linked glycosylation in the ER, disulfide bond formation by folding intermediates is essential for the maturation of proteins destined for the secretory pathway (Helenius and Aebi, 2001; Ellgaard et al., 2018; Marinko et al., 2019). Misfolded proteins can utilize free thiols to form non-native disulfide bonds with unpaired cysteine residues on chaperones thereby instigating their retention within the ER (Anelli et al., 2003). Using non-reducing PAGE and the thiol-reactive reagents BMH and mPEG-MAL-5000, we found that all FKRP mutations form high molecular weight aggregates thereby reducing the levels of the correctly folded FKRP monomer. These aggregates result from the formation of intra- and intermolecular disulfide bonds between adjacent FKRP molecules and may also involve interactions with ER-resident chaperones (see below). Furthermore, wild type FKRP and disease-causing FKRP variants appear to differentially associate with a range of ER-resident chaperones (Figure 5). Thus, interactions between FKRP mutants and the ER-resident chaperones, such as calnexin, the protein disulfide isomerase ERp72 and GRP94 (Table 2), may regulate the folding of the nascent protein and transit through the early secretory pathway. Missense mutants that fail to adopt native conformations that are retained in the ER may undergo ERAD thereby reducing the levels of functional protein that can be trafficked to the Golgi apparatus (Esapa et al., 2005). Although most *FKRP* mutations led to ER-retention of the mutant protein (Esapa et al., 2002; Esapa et al., 2005; Keramaris-Vrantsis et al., 2007) misfolded variants such as p.C318Y can traffic to the Golgi apparatus (Supplementary Figure S1) as described previously (Dolatshad et al., 2005; Henriques et al., 2019). Henriques and colleagues found that p.C318Y was catalytically inactive in assays for dystroglycan glycosylation implying that certain misfolded non-functional FKRP variants can evade ER-associated quality control checkpoints and traffic to the Golgi apparatus (Henriques et al., 2019). By contrast, the LGMDR9 mutation p.W231C is partially retained within the ER of COS-7 cells and myotubes but clearly adopts non-native conformations (Figure 3 Figure 4A and Supplementary Figure S1). Thus, ER-retention is not necessarily a proxy for misfolding nor is it correlated with disease severity but may indicate that some FKRP mutants may undergo ERAD as demonstrated previously (Esapa et al., 2005).

Having found that mixed disulfide bond formation may participate in the misfolding of some FKRP variants, we used site-directed mutagenesis to examine the role of each of the eight cysteine residues on the synthesis of FKRP. Previous studies suggested that FKRP is a homodimer, covalently bonded through the single cytoplasmic cysteine residue C6 (Alhamidi et al., 2011). Using non-reducing PAGE our data suggest that FKRP does not form a covalently bonded homodimer but can be crosslinked to an adjacent FKRP molecule through C6 (Figure 1A; Figure 2B). These findings are also supported by the crystal structure where protomeric dimers are not formed by intramolecular disulfide bonds (Kuwabara et al., 2020). Chronic treatment of cells with the reducing agent DTT at concentrations that do not adversely affect the functioning of the secretory pathway affected the formation of monomeric p.W231C and p.P448L in the ER (Braakman et al., 1992). Under non-reducing PAGE conditions, DTT-treatment resulted in the rapid appearance of the monomeric FKRP allied with reduction in levels of the high molecular weight aggregates that contained misfolded FKRP (Figure 4A). Preliminary observations on p.W231C may suggest that DTT treatment also resulted in trafficking of mutant FKRP from the ER to the Golgi apparatus (Figure 4C). Whether DTT treatment can improve the folding of disease-associated FKRP variants has not been established. These findings further demonstrate the involvement of non-native disulfide bond formation in the misfolding and ER-retention of some FKRP mutants and may provide potential targets for therapeutic intervention (see below).

In addition to the biochemical techniques described above, we used FRAP to study the dynamic properties of FKRP and a panel of disease-associated mutations in living cells. FRAP analysis has been used to demonstrate misfolding of the temperature sensitive VSV-G protein (Nehls et al., 2000) and the common cystic fibrosis variant, p.Phe508del (Haggie et al., 2002). Using the same methodology, we have found that the behavior of many of the disease-associated mutants, such as p.P448L, differs dramatically from that of the wild type FKRP in both COS-7 cells and C2C12 myoblasts (Figure 3 and Supplementary Figure S2). For example, following ATP-depletion most of the mutants studied showed a large reduction in their diffusion coefficients and mobile fraction (Figure 3; Table 2). ATP depletion had a relatively modest effect on p.L276I, a partially misfolded protein, and no apparent effect on wild type FKRP. Remarkably, p.P448L mobility was restored with DTT, suggesting that illegitimate disulfide bond formation may participate in the misfolding process as described for other proteins (Nehls et al., 2000). Interestingly, p.C318Y, that can be trafficked to the Golgi apparatus, also behaved like a typical misfolded protein in ATP-depleted cells (Figure 3).

We also used structural modelling and molecular dynamics simulations to examine the site and effect of different missense mutations on the 3D structure of FKRP (Figure 6). It is noteworthy that attempts to purify other FKRP mutants (including p.L276I and p.S221R) by Kuwabara and colleagues for structure/function studies were unsuccessful because these proteins were poorly secreted from transfected cells and could not be purified (Kuwabara et al., 2020). Most FKRP variants that were modelled had subtle but significant effects on the local environment of FKRP and could result in misfolding of the monomer affecting formation of the functional protomeric dimer. The most dramatic change involved p.C318Y that forms part of the Zn^2+^ finger loop of FKRP. The cation is required to stabilize the loop formed by G288-C318 (Kuwabara et al., 2020). The bulky p.C318Y substitution may lead to local destabilization in the vicinity of the Zn^2+^ finger loop that could reshape the active site of the catalytic domain (Figure 6E). The p.P448L substitution that causes CMD is arguably the most studied FKRP variant in the literature (Ortiz-Cordero et al., 2021a). Mouse models carrying this variant develop a severe muscular dystrophy with almost absent functional α-dystroglycan glycosylation (Chan et al., 2010). Although p.P448L retains significant enzymatic activity it behaves as a typical misfolded protein in all the assays presented herein (Tucker et al., 2018). Structurally, P448 is located at the solvent interface in the FKRP structure and is unlikely to directly alter the enzymatic function of the protein (Figure 6G). The p.P448L substitution could form a hydrophobic patch on the protein that may lead to local misfolding.

Taken together, our data highlight the idiosyncratic behavior of the different disease-associated FKRP missense mutations and suggest that misfolding plays an important role in the molecular pathology of the range of disorders caused by *FKRP* mutations. Mechanistically the misfolding of FKRP variants results in the formation of reversible, non-native disulfide bonds potentially involving ER-resident chaperones such as calnexin and ERp72. The discovery that reducing agents can improve protein folding and enhance intracellular transport of different variants may have a therapeutic potential for treating FKRP-deficient muscular dystrophies. Indeed, structurally informed small molecule “correctors” that affect the biosynthesis and trafficking of mutant CFTR through the secretory pathway are now used to treat cystic fibrosis (Middleton et al., 2019). It is therefore tempting to speculate that compounds that assist the folding of disease-associated variants could be used to treat FKRP-deficient muscular dystrophies.

## 4 Materials and methods

### 4.1 Molecular biology

The “Quick-Change” site-directed mutagenesis kit (Agilent) was used to introduce point mutations in the FKRP cDNA (Esapa et al., 2002). All constructs were verified by Sanger sequencing. The primers used to make the panel of FKRP mutants are shown in the Supplementary Table S1. Expression constructs for live cell imaging were generated from full length FKRP (wild type and those carrying single missense mutations) by PCR amplification using Pfu Ultra (Agilent) with the forward primer 5′-CGGGAA​TTCTGC​CCA​TGC​GGC​TCA​CCC​GCT​GC and the reverse primer 5′-CCGGAA​TTCGAC​CGC​CTG​TCA​AGC​TTA​AGA​G (*EcoR*I sites underlined). PCR products were sub-cloned into the *EcoR*I site of pEYFP-N1 vector (Clontech) fusing the fluorescent protein tag to the luminal C-terminus of FKRP. Myc-tagged constructs were generated as described previously (Esapa et al., 2005).

### 4.2 Cell culture and transfection

COS-7, HEK293T and C2C12 cells were grown at 37°C and 5% (*v/v*) CO_2_ using Dulbecco’s modified Eagle’s medium (DMEM) supplemented with Glutamax (Thermofisher), 10% (*v/v*) fetal calf serum and penicillin/streptomycin (Sigma). Cells grown in six-well plates, with and without cover slips, were transfected with 1 μg of each expression construct using Fugene 6 (Roche) according to the manufacturer’s instructions. Myotubes were differentiated from semi-confluent C2C12 myoblasts by culture in low serum media (DMEM supplemented with 2% *v/v* horse serum, Glutamax and penicillin/streptomycin) on collagen-coated cover slips for 5 days. Commercial antibodies were purchased from BD Bioscience (GM130), Stressgen (protein disulfide isomerase, PDI) and Invitrogen (Alexa Fluor 488 and 568). The anti-FKRP STEM-819 antibody used in this study has been described previously (Esapa et al., 2005).

### 4.3 Biochemical techniques

The amine reactive, membrane permeable crosslinker, dithiobis [succinimidyl propionate] (DSP) was used to crosslink proteins prior to polyacrylamide gel electrophoresis (PAGE) and for immunoaffinity purification. For *in vivo* crosslinking, live transfected HEK293T cells were treated with 2 mM DSP for 10 min prior to quenching with 50 mM Tris pH7.4 and lysis in PBS containing 1% (*v/v*) Triton X-100 and 10 mM iodoacetamide to prevent disulfide bond exchange after the cells were lysed. DSP-crosslinks were cleaved by incubation in SDS-PAGE sample buffer containing DTT. For bismaleimidohexane (BMH, 1.3 nm spacer) crosslinking, transfected cells were lysed using PBS/1% (*v/v*) Triton X-100 in the presence of the homobifunctional thiol-reactive crosslinker BMH (Pierce) at a final concentration of 1 mM. The reaction was incubated for 30 min at room temperature before quenching by adding an equal volume of 2 × SDS-PAGE sample buffer containing 20 mM DTT. For PEGylation, transfected cells were lysed with PBS/1% (*v/v*) Triton X-100 containing the sulfhydryl alkylating reagent mPEG-MAL-5000 (Nektar) at a final concentration of 3 mM (Braakman et al., 2017). The reaction was incubated at room temperature for 45 min before quenching with 20 mM DTT. Non-reducing and denaturing PAGE were performed as described previously (Esapa et al., 2007). Proteins were visualized using enhanced chemiluminescence (Pierce) or using the LI-COR Odyssey Imaging System. The relative levels of FKRP monomer and high molecular weight aggregates on Western blots were quantified using Fiji/ImageJ (Schindelin et al., 2012; Gallo-Oller et al., 2018).

### 4.4 Live cell microscopy and fluorescence recovery after photobleaching (FRAP)

FRAP studies on the intracellular mobility of FKRP and mutants thereof were conducted as described previously (Esapa et al., 2007; Pooler and McIlhinney, 2007). Briefly, COS-7 cells or C2C12 myoblasts were grown on cover slips and transfected with EYFP-tagged FKRP constructs and mutants thereof. Twenty-4 hours after transfection the cells were washed with Hank’s buffered salt solution containing 0.33 mM D-glucose and treated with 1 μM brefeldin A (Sigma), to inhibit protein transport from the ER to the Golgi apparatus, for 1 h prior to FRAP. Brefeldin A was present with the Hank’s buffered saline for the duration of the FRAP experiments. ATP depletion was carried out by adding 2-deoxyglucose (0.5 mM) and 0.02% (*w/v*) sodium azide to the cells for 30 min, as described by Nehls et al. (2000) in glucose free Hank’s buffered saline. Preliminary experiments showed that the effect of 2-deoxyglucose on the cells was dose dependent with the lowest effective concentration being 0.5 mM. ATP-depleted cells were not harmed by the treatment, as they showed full fluorescence recovery when replaced into tissue culture medium for 1 h after the experiments were completed. Photobleaching was carried out using the 514 nm Argon laser line of an LSM 510 Zeiss confocal microscope at 75% of full power equivalent to 17 mW, recovery was monitored at 1% of this laser output at 30°C. The bleached regions of interest were generally 50 pixels in diameter and seven iterations were performed to fully photobleach the region of interest. Unbleached regions of the same cell were used to control for photobleaching during the recovery period, though this was generally minimal. Diffusion coefficients and the mobile fraction for each experiment were calculated as described by Barak and colleagues (Barak et al., 1997).

### 4.5 Mass spectrometry

HEK293T cells expressing myc-tagged FKRP and mutants thereof were incubated with DSP before lysis as described above. Myc-tagged proteins (and their associated chaperones) were purified from whole cell extracts using anti-myc 9E10 conjugated beads as described previously (Locke et al., 2009). Proteins extracted from untransfected cells were processed as described above and used as a control for mass spectrometry and western blotting. Eluted proteins were resolved on Novex polyacrylamide gradient gels (Thermofisher), stained and processed for mass spectrometry as described previously (Benson et al., 2017). Briefly, individual Coomassie-stained bands were excised, digested with trypsin and analyzed by Fourier-transform ion cyclotron resonance mass spectrometry (FTICR-MS) at the Advanced Mass Spectrometry Facility at the University of Birmingham (Waite et al., 2016; Benson et al., 2017). Data analysis and protein identification were performed using Sequest software (Proteome Discoverer, Thermofisher). Proteins that co-purified with wild type and mutant FKRP were validated by western blotting using appropriate antibodies.

### 4.6 Structural modelling and molecular dynamics

The 3D structure of FKRP deposited as PDB:6KAM (https://www.rcsb.org/structure/6KAM) was used to model each missense mutation (Kuwabara et al., 2020). Point mutations were introduced in to the protomeric dimer AB of the starting model while energy minimisation and molecular dynamics were performed using Modeller (Sali and Blundell, 1993). Molecular dynamics was applied for 200 iterations with stereochemistry optimisation at a local level. Optimisation of structures was run in two passes, first with mutated residue atoms then including non-bonded neighbouring atoms of mutated residues. Interface energy estimates in a tetramer were made with PISA (Krissinel and Henrick, 2007). Graphical representations of the models were prepared with PYMOL (http://www.pymol.org/pymol): The PyMOL Molecular Graphics System, Version 1.2r3pre, Schrödinger, LLC).

## Data Availability

The original contributions presented in the study are included in the article/Supplementary Material, further inquiries can be directed to the corresponding author.
