# Supplementary material for: Misfolding of fukutin-related protein (FKRP) variants in congenital and limb girdle muscular dystrophies
Source: Front Mol Biosci. 2023 Dec 7;10:1279700. doi: 10.3389/fmolb.2023.1279700 (PMC10755465; doi:10.3389/fmolb.2023.1279700)

**SUPPLEMENTARY MATERIALS**

**Supplementary Table S1 Mutagenic Primers**

Site-directed mutagenesis was used to introduce point mutations in the FKRP cDNA. Where appropriate, the original citations for each mutation are shown in Table 1. Mutagenic primers are shown below. Altered nucleotides are underlined.

| Variant | Forward primer | Reverse primer |
| --- | --- | --- |
| C6A | GCGGCTCACCCGCGCCTGGGCTGCCCTGGC | GCCAGGGCAGCCCAGGCGCGGGTGAGCCGC |
| C168A | CCAACCCAGCGCGGGCCCTAGCTCTGAACG | CGTTCAGAGCTAGGGCCCGCGCTGGGTTGG |
| C191A | CCAGCGCGCCCCGCGCCGACGCTTTGGATG | CATCCAAAGCGTCGGCGCGGGGCGCGCTGG |
| C289A | CTAGAGTGGTTTGGCGCCAGCAAGGAGAGCG | CGCTCTCCTTGCTGGCGCCAAACCACTCTAG |
| C296A | GGAGAGCGCGCGCGCCTTCGGTACGGTGGC | GCCACCGTACCGAAGGCGCGCGCGCTCTCC |
| C317A | GCTGGACCCCACCTGCTTGCCTGCGCGCGC | GCGCGCGCAGGCAAGCAGGTGGGGTCCAGC |
| C318Y | GGACCCCACCTTGTTACCTGCGCGCGCTGC | GCAGCGCGCGCAGGTAACAAGGTGGGGTCC |
| C375A | GAGGACGTGGGCAACGCCGAGCAGTTGCGGG | CCCGCAACTGCTCGGCGTTGCCCACGTCCTC |
| S221R | GCTGGCCACCAGACTCTTCCTACAGACCGCC | GGCGGTCTGTAGGAAGAGTCTGGTGGCCAGC |
| W231C | CCCTGCGCGGCTGCGCAGTGCAGCTGCTG | CAGCAGCTGCACTGCGCAGCCGCGCAGGG |
| Y307N | GCGACACACCCGCCAACCTGTATGAGGGCC | GGCCCTCATACAGGTTGGCGGGTGTGTCGC |
| Y309C | CGACACACCCGCCTACCTGTGTGAGGGCCGCTGGACC | GGTCCAGCGGCCCTCACACAGGTAGGCGGGTGTGTCG |
| P316T | GGGCCGCTGGACCCCAACTTGTTGCCTGC | GCAGGCAACAAGTTGGGGTCCAGCGGCCC |
| V405L | GCGACTTCTTCCGACTACAGTACAGTGAG | CTCACTGTACTGTAGTCGGAAGAAGTCGC |
| A455D | GTCCCCCTGCCCTTTGACGGTTTCATGGCACAGGC | GCCTGTGCCATGAAACCGTCAAAGGGCAGGGGGAC |
| P488L | GCACTTCCTGCAGCTACTTGTCCCCCTGC | GCAGGGGGACAAGTAGCTGCAGGAAGTGC |

**Supplementary Table S2 Protein identification and peptides from FKRP-IAP**

| **Protein identity** | **Unique peptides** | **XC score** |
| --- | --- | --- |
| **NP_003290.1**/*HSP90B1* (heat shock protein 90 beta family member 1)/GRP94 | R.KEAESSPFVER.L  K.SGTSEFLNK.M  K.EESDDEAAVEEEEEEKKPK.T  K.TETVEEPM#EEEEAAK.E  K.TETVEEPM#EEEEAAKEEK.E  K.EAESSPFVER.L  K.DISTNYYASQK.K  K.IYFM#AGSSR.K  K.DISTNYYASQK.K  K.TFEINPR.H  R.SGYLLPDTK.A  K.NLLHVTDTGVGM#TR.E  K.NLLHVTDTGVGM#TR.E  R.RVFITDDFHDM#M#PK.Y  K.YLNFVK.G  K.FAFQAEVNR.M  K.FAFQAEVNR.M  R.GLFDEYGSK.K  R.VFITDDFHDM#M#PK.Y  R.LSLNIDPDAK.V  R.GTTITLVLK.E  K.LIINSLYK.N  R.FQSSHHPTDITSLDQYVER.M  R.FQSSHHPTDITSLDQYVER.M  K.GVVDSDDLPLNVSR.E  K.SILFVPTSAPR.G  R.EEEAIQLDGLNASQIR.E  K.EEASDYLELDTIK.N  R.LISLTDENALSGNEELTVK.I  R.LISLTDENALSGNEELTVK.I  R.TDDEVVQREEEAIQLDGLNASQIR.E | 260.30 |
| **NP_005337.1**/*HSPA1B* (heat shock protein family A (Hsp70) member 1B)/HSP70.2 | R.LVNHFVEEFK.R  K.HWPFQVINDGDKPK.V  R.LVNHFVEEFKR.K  K.HWPFQVINDGDKPK.V  R.LVNHFVEEFK.R  K.AQIHDLVLVGGSTR.I  R.LVNHFVEEFKR.K  R.LVNHFVEEFK.R  K.FGDPVVQSDM#K.H  K.FGDPVVQSDM#K.H  K.AQIHDLVLVGGSTR.I  R.LVNHFVEEFK.R  K.NQVALNPQNTVFDAK.R  K.HWPFQVINDGDKPK.V  K.HWPFQVINDGDKPK.V  K.AQIHDLVLVGGSTR.I  K.FGDPVVQSDM#K.H  R.LVNHFVEEFK.R  R.LVNHFVEEFKR.K  R.LVNHFVEEFK.R  R.LVNHFVEEFKR.K  K.FGDPVVQSDM#K.H  R.LVNHFVEEFK.R  K.NQVALNPQNTVFDAK.R  K.AQIHDLVLVGGSTR.I  R.LVNHFVEEFK.R  K.NQVALNPQNTVFDAK.R  K.GGSGSGPTIEEVD.-  K.GGSGSGPTIEEVD.-  K.GGSGSGPTIEEVD.-  K.NQVALNPQNTVFDAK.R  K.NQVALNPQNTVFDAK.R | 68.25 |
| **NP_005518.2**/*HSPA1L* (heat shock protein family A (Hsp70) member 1 like)/HPS70-1L | K.DAGVIAGLNVLR.I  K.DAGVIAGLNVLR.I  K.ATAGDTHLGGEDFDNR.L  K.ATAGDTHLGGEDFDNR.L  K.NALESYAFNM#K.S  K.DAGVIAGLNVLR.I  K.NALESYAFNM#K.S  K.AKIHDIVLVGGSTR.I  K.ATAGDTHLGGEDFDNR.L  K.AKIHDIVLVGGSTR.I  K.NALESYAFNM#K.S  K.AKIHDIVLVGGSTR.I  K.DAGVIAGLNVLR.I  K.NALESYAFNM#K.S  K.DAGVIAGLNVLR.I  K.NALESYAFNM#K.S  K.ATAGDTHLGGEDFDNR.L  K.DAGVIAGLNVLR.I  K.DAGVIAGLNVLR.I  K.DAGVIAGLNVLR.I | 40.20 |
| **NP_009057.1**/*VCP*(valosin containing protein)/p97 | R.VVRNNLR.V  R.EVDIGIPDATGR.L  R.GILLYGPPGTGK.T | 30.14 |
| **NP_005339.2**/*HSP90AA1* (heat shock protein 90 alpha family class A member 1)/HSP90A | K.HIYYITGETK.D  K.DQVANSAFVER.L  K.FYEQFSK.N  R.ELISNSSDALDK.I  R.ELISNSSDALDK.I  R.YYTSASGDEM#VSLK.D  K.FYEQFSK.N  R.TLTIVDTGIGM#TK.A  K.ELHINLIPNK.Q  K.HLEINPDHSIIETLR.Q  R.ALLFVPR.R  R.NPDDITNEEYGEFYK.S  R.NPDDITNEEYGEFYK.S  R.TLTIVDTGIGMTK.A  R.RAPFDLFENR.K  R.RAPFDLFENR.K  R.APFDLFENR.K  R.APFDLFENR.K | 126.22 |
| **NP_031381.2**/*HSP90AB1* (heat shock protein 90 alpha family class B member 1)/HSP90B | R.YESLTDPSK.L  K.TKPIWTR.N  K.YIDQEELNK.T  K.EQVANSAFVER.V  R.RLSELLR.Y  K.EDQTEYLEER.R  K.EQVANSAFVER.V  R.YESLTDPSKLDSGK.E  R.YESLTDPSKLDSGK.E  K.SIYYITGESK.E  R.ELISNASDALDK.I  R.YHTSQSGDEM#TSLSEYVSR.M  K.EGLELPEDEEEK.K  K.IDIIPNPQER.T  K.SIYYITGESK.E  K.VILHLKEDQTEYLEER.R  K.HLEINPDHPIVETLR.Q  K.VILHLKEDQTEYLEER.R  R.TLTLVDTGIGM#TK.A  K.HFSVEGQLEFR.A  K.HFSVEGQLEFR.A  K.IDIIPNPQER.T  K.SLTNDWEDHLAVK.H  K.SLTNDWEDHLAVK.H  K.HFSVEGQLEFR.A  R.NPDDITQEEYGEFYK.S  K.ADLINNLGTIAK.S  R.GVVDSEDLPLNISR.E  R.RAPFDLFENK.K  R.TLTLVDTGIGMTK.A  R.ALLFIPR.R  R.NPDDITQEEYGEFYK.S  R.APFDLFENK.K  R.APFDLFENK.K  K.HSQFIGYPITLYLEK.E | 238.21 |
| **NP_005337.1**/*HSPA1B* (heat shock protein family A (Hsp70) member 1B)/HSP70.2 | R.LVNHFVEEFK.R  K.AQIHDLVLVGGSTR.I  R.LVNHFVEEFKR.K  K.HWPFQVINDGDKPK.V  R.LVNHFVEEFK.R  K.NQVALNPQNTVFDAK.R  R.LVNHFVEEFK.R  K.AQIHDLVLVGGSTR.I  K.NQVALNPQNTVFDAK.R  R.LVNHFVEEFK.R  K.NQVALNPQNTVFDAK.R  K.HWPFQVINDGDKPK.V  R.LVNHFVEEFKR.K  K.GGSGSGPTIEEVD.- | 60.22 |
| **NP_005338.1**/*HSPA5* (heat shock protein family A (Hsp70) member 5)/BIP, GRP78 | K.VYEGERPLTK.D  K.M#KETAEAYLGK.K  K.M#KETAEAYLGK.K  K.VLEDSDLK.K  R.LTPEEIER.M  K.ETAEAYLGK.K  K.TKPYIQVDIGGGQTK.T  K.TKPYIQVDIGGGQTK.T  R.TWNDPSVQQDIK.F  K.TKPYIQVDIGGGQTK.T  K.DAGTIAGLNVM#R.I  K.KSQIFSTASDNQPTVTIK.V  K.LYGSAGPPPTGEEDTAEKDEL.-  K.NQLTSNPENTVFDAK.R  K.KKELEEIVQPIISK.L  K.KVTHAVVTVPAYFNDAQR.Q  K.PYIQVDIGGGQTK.T  K.KVTHAVVTVPAYFNDAQR.Q  K.KVTHAVVTVPAYFNDAQR.Q  K.SQIFSTASDNQPTVTIK.V  K.KSDIDEIVLVGGSTR.I  K.SQIFSTASDNQPTVTIK.V  K.KSDIDEIVLVGGSTR.I  K.VTHAVVTVPAYFNDAQR.Q  R.ITPSYVAFTPEGER.L  K.VTHAVVTVPAYFNDAQR.Q  K.DAGTIAGLNVMR.I  K.KSDIDEIVLVGGSTR.I  R.NELESYAYSLK.N  R.ITPSYVAFTPEGER.L  R.AKFEELNM#DLFR.S  R.AKFEELNM#DLFR.S  R.IINEPTAAAIAYGLDKR.E  K.VTHAVVTVPAYFNDAQR.Q  K.ELEEIVQPIISK.L  R.IINEPTAAAIAYGLDKR.E  K.SDIDEIVLVGGSTR.I  R.NELESYAYSLK.N  R.ITPSYVAFTPEGER.L  K.TFAPEEISAM#VLTK.M  K.FEELNM#DLFR.S  K.IEWLESHQDADIEDFK.A  R.ITPSYVAFTPEGER.L  R.IINEPTAAAIAYGLDKR.E  K.DNHLLGTFDLTGIPPAPR.G  R.IEIESFYEGEDFSETLTR.A | 286.22 |
| **NP_004125.3**/*HSPA9* (heat shock protein family A (Hsp70) member 9)/GRP75 | K.M#KETAENYLGHTAK.N  K.LFEM#AYK.K  R.QAVTNPNNTFYATK.R  R.ETGVDLTKDNM#ALQR.V  R.EQQIVIQSSGGLSK.D  K.KSQVFSTAADGQTQVEIK.V  K.SQVFSTAADGQTQVEIK.V  R.TTPSVVAFTADGER.L  R.EQQIVIQSSGGLSKDDIENM#VK.N  K.ERVEAVNM#AEGIIHDTETK.M  R.VEAVNM#AEGIIHDTETK.M  K.VQQTVQDLFGR.A  K.DAGQISGLNVLR.V  K.SDIGEVILVGGM#TR.M  K.DAGQISGLNVLR.V  R.VINEPTAAALAYGLDKSEDK.V  K.S*DIGEVILVGGMT*RM#PK.V  K.S*DIGEVILVGGM#T*RMPK.V  K.LYSPSQIGAFVLM#K.M  R.AQFEGIVTDLIR.R  K.STNGDTFLGGEDFDQALLR.H  K.LLGQFTLIGIPPAPR.G  R.AQFEGIVTDLIR.R | 204.25 |
| **NP_068814.2**/*HSPA2* (heat shock protein family A (Hsp70) member 2)/HPS70-2 | K.VEIIANDQGNR.T  K.STAGDTHLGGEDFDNR.M  R.TTPSYVAFTDTER.L  R.IINEPTAAAIAYGLDKK.G  K.LLQDFFNGK.E  R.IINEPTAAAIAYGLDK.K  R.ARFEELNADLFR.G  R.IINEPTAAAIAYGLDK.K  R.FEELNADLFR.G  R.IINEPTAAAIAYGLDK.K | 80.23 |
| **NP_057376.1**/*TRAP1* (TNF receptor associated protein 1)/HSP75 | R.EGIVTATEQEVK.E  R.ELISNASDALEK.L  R.ELGSSVALYSR.K  R.ELLQESALIR.K  R.AQLLQPTLEINPR.H  R.GVVDSEDIPLNLSR.E | 58.17 |
| **NP_694881.1**/*HSPA8* (heat shock protein family A (Hsp70) member 8)/HSC70 | K.SQIHDIVLVGGSTR.I  R.M#VNHFIAEFK.R  R.M#VNHFIAEFK.R  K.DAGTIAGLNVLR.I  K.SFYPEEVSSM#VLTK.M  K.SFYPEEVSSM#VLTK.M  K.TVTNAVVTVPAYFNDSQR.Q | 50.19 |
| **NP_004902.1**/*PDIA4* (protein disulfide isomerase family A member 4)/ERp72 | K.VDATAETDLAK.R  K.VSQGQLVVM#QPEK.F  K.VSQGQLVVM#QPEK.F  K.IDATSASVLASR.F  K.IDATSASVLASR.F  K.TFDSIVM#DPK.K  K.TFDSIVM#DPK.K  K.RFDVSGYPTLK.I  R.FDVSGYPTIK.I  R.FDVSGYPTLK.I  K.FAM#EPEEFDSDTLR.E  K.FAM#EPEEFDSDTLR.E  K.DLGLSESGEDVNAAILDESGKK.F | 88.19 |
| **NP_001737.1**/*CANX* (calnexin)/CNX | K.AEEDEILNRS#PR.N  K.TPELNLDQFHDK.T  K.TPELNLDQFHDK.T  K.LHFIFR.H  K.TPYTIM*FGPDK.C  R.GTLSGWILSK.A  R.IVDDWANDGWGLKK.A  R.GTLSGWILSK.A | 60.17 |

**Supplementary Figure Legends**

**Supplementary Figure S1 Immunolocalization of EYFP-tagged FKRP constructs in C2C12 myotubes.**

C2C12 myoblasts were transfected with different EYFP-tagged FKRP as indicated. Myotubes that formed after six days differentiation were fixed and [permeabilized](https://www.google.com/search?client=safari&rls=en&q=permeabilized&spell=1&sa=X&ved=0ahUKEwiVpril_rvbAhVNJVAKHU0xAhkQkeECCCYoAA) and labelled with either GM130 (Golgi apparatus) or PDI (protein disulfide isomerase, ER). Wild type FKRP precisely co-localises with GM130 in the perinuclear region of transfected myotubes. By contrast, all FKRP mutants are, to a greater or lesser extent, detectible in the ER (co-localising with PDI). Notably, p.L276I and p.C318Y clearly traffic to the Golgi apparatus. Scale bar is 20µm.

**Supplementary Figure S2 FRAP analysis of FKRP, p.L276I and p.P448L in C2C12 myoblasts.**

FRAP analysis and measurement of diffusion rates in the ER were conducted as described in Materials and Methods. Fluorescence recovery in D-glucose-containing media is shown in red and recovery in ATP-depleted medium is shown in green. For P448L, the fluorescence recovery in the presence of 5mM DTT (with ATP depletion) is shown by the blue line. Each point represents the mean fluorescence recovery from analysis of at least 10 cells + SEM. Similar results were obtained in three separate experiments.

**Supplementary Figure S1**


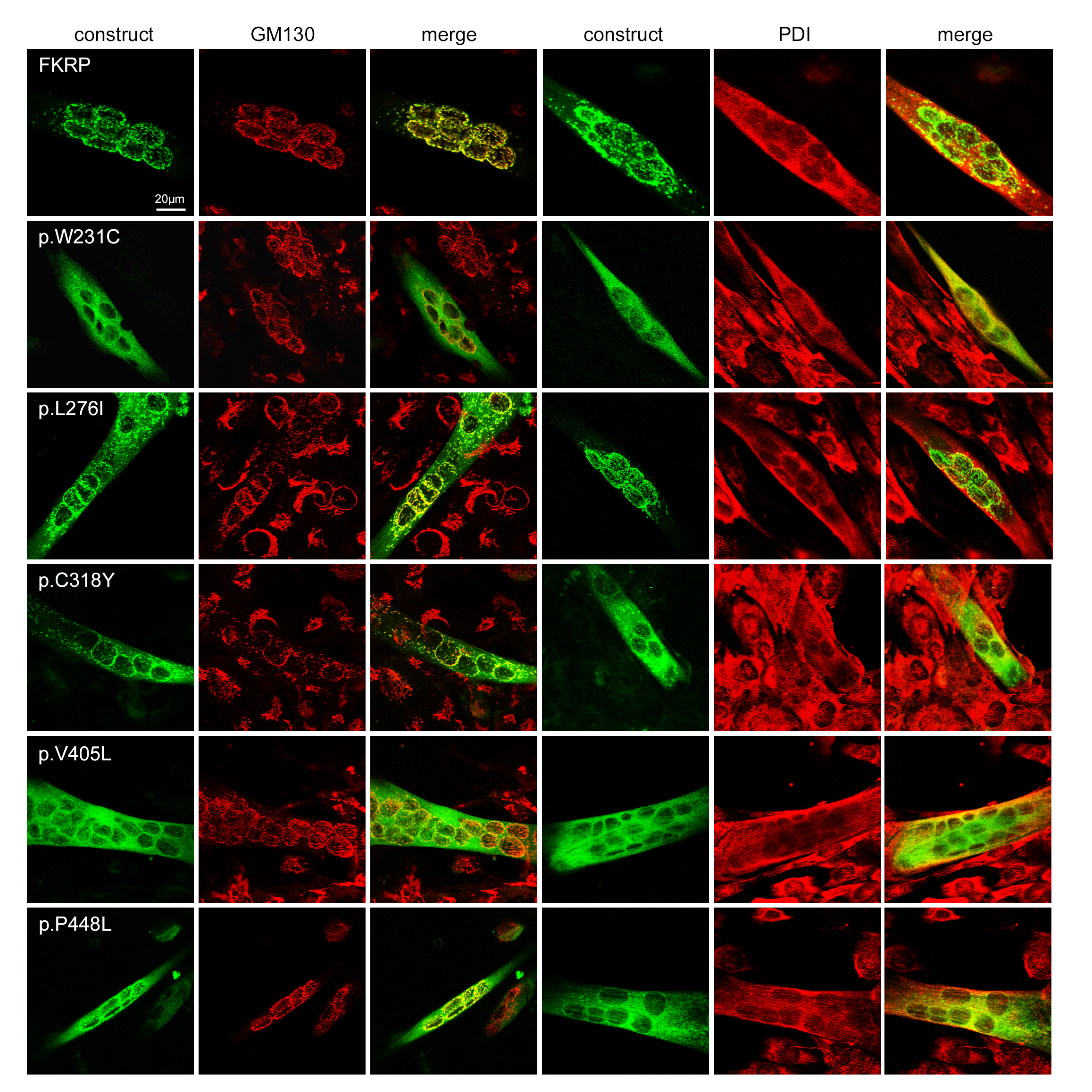


**Supplementary Figure S2**


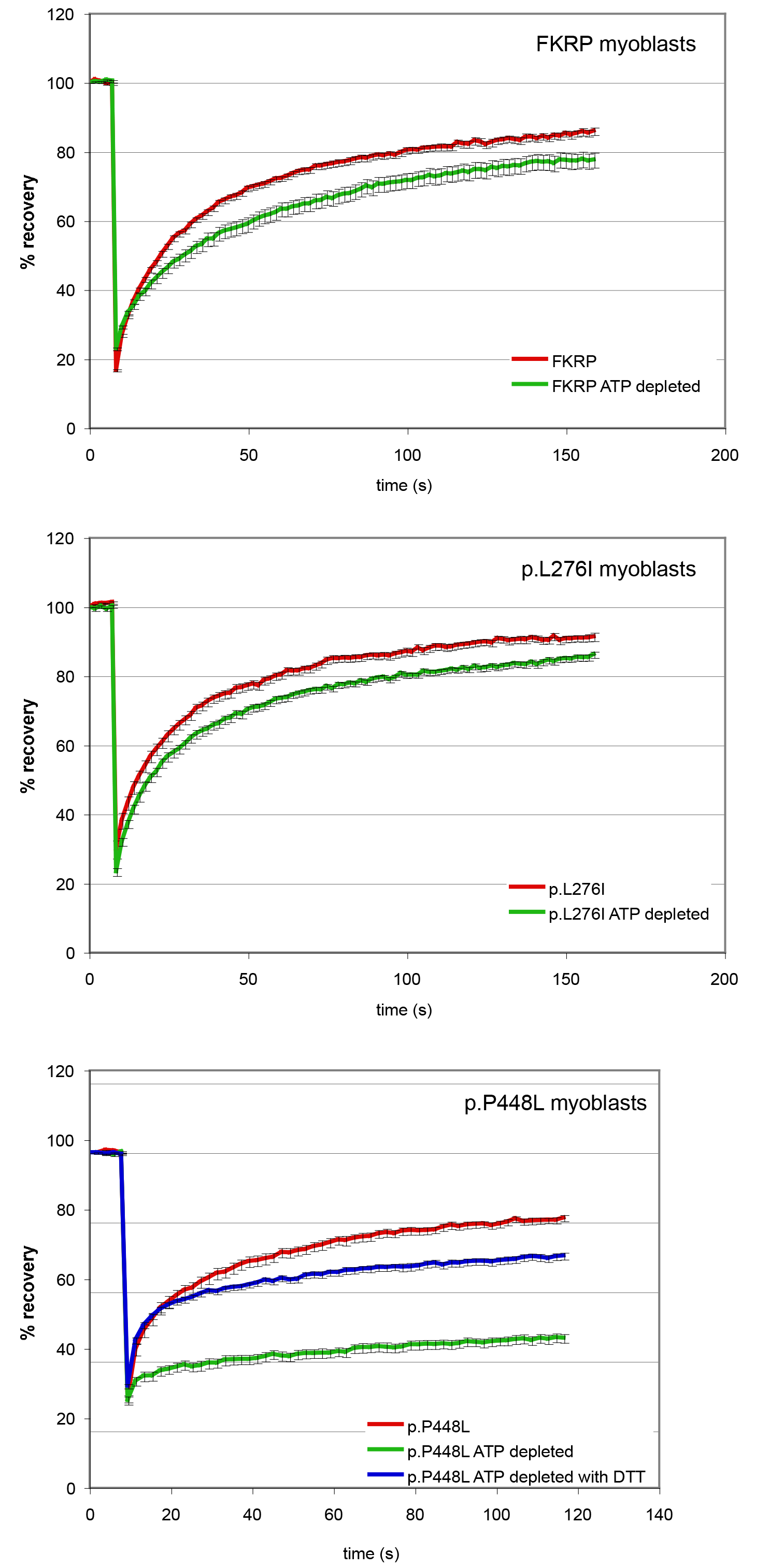

Supplement: Supplementary file 1 [file Table1.DOCX]
